# ADAMTS7, a target in atherosclerosis, cooperates with its homolog ADAMTS12 to protect against myxomatous valve degeneration

**DOI:** 10.1016/j.jmccpl.2025.100288

**Published:** 2025-02-22

**Authors:** Timothy J. Mead, Sumit Bhutada, Niccolò Peruzzi, Janet Adegboye, Deborah E. Seifert, Elisabeth Cahill, Jeanne Drinko, Eoin Donnellan, Anu Guggiliam, Zoran Popovic, Brian Griffin, Karin Tran-Lundmark, Suneel S. Apte

**Affiliations:** a Department of Biomedical Engineering, Cleveland Clinic Lerner Research Institute, Cleveland, OH, USA; b Department of Pediatrics, Case Western Reserve University School of Medicine, Cleveland, OH, USA; c University Hospitals Rainbow Babies and Children's Hospital, Cleveland, OH, USA; d Department of Experimental Medical Science and Wallenberg Center for Molecular Medicine, Lund University, Lund, Sweden; e Department of Cardiovascular Medicine, Cleveland Clinic Lerner Research Institute, Cleveland, OH, USA; f Department of Cellular and Molecular Medicine, Cleveland Clinic Lerner Research Institute, Cleveland, OH, USA; g The Pediatric Heart Center, Skane University Hospital, Lund, Sweden

**Keywords:** ADAMTS, Extracellular matrix, Metalloproteinase, Proteoglycan, Myxomatous valve, Heart valve, Regurgitation, Hypertrophy, Fibrosis

## Abstract

The physiological roles of the metalloprotease-proteoglycan ADAMTS7, a drug target in atherosclerosis and vascular restenosis, and its homolog ADAMTS12, are undefined in the cardiovascular system. The objective of the present work was to investigate their roles in mice with genetic inactivation of both proteases and in relation to the resulting valve defects, to define their proteolytic activities in the matrisome. Here, we demonstrate that *Adamts7* and *Adamts12* are co-expressed in heart valves and each buffers inactivation of the other by compensatory upregulation. Leaflets of *Adamts7*^−/−^;*Adamts12*^−/−^ aortic valves, but not the respective single mutants, were abnormally shaped at birth, with progressively severe disorganization and enlargement occurring thereafter. Doppler echocardiography showed that *Adamts7*^−/−^;*Adamts12*^−/−^ mice had stenotic and regurgitant aortic valves. We investigated ADAMTS7 and ADAMTS12 substrates relevant to the valve matrisome in secretome libraries from *Adamts7*^−/−^;*Adamts12*^−/−^ cells using the N-terminomics technique Terminal Amine Isotopic Labeling of Substrates (TAILS). Although ADAMTS7 and ADAMTS12 shared several extracellular matrix (ECM) substrates, cleavage sites and sequence preference for each protease were distinct. *Adamts7*^−/−^;*Adamts12*^−/−^ valve leaflets showed accumulation of several of the identified ECM substrates, including periostin, a matricellular protein crucial for cardiac valve homeostasis. We conclude that the myxomatous degeneration in *Adamts7*^−/−^;*Adamts12*^−/−^ valve leaflets reflects a complex disturbance of ECM proteostasis with accumulation of multiple ADAMTS7 and ADAMTS12 ECM substrates, and perturbation of regulatory pathways with roots in ECM, such as TGFβ signaling, which was increased in the mutant valves.

## Highlights

• ADAMTS7 and ADAMTS12 are matrix-degrading proteases co-expressed in heart valves.
• Mice lacking both ADAMTS7 and ADAMTS12 have incompetent, myxomatous heart valves.
• N-terminomics provided de novo identification of ADAMTS7 and ADAMTS12 substrates.
• Valve defects in the mutant mice reflect ECM accumulation and TGFβ dysregulation.

## Graphical abstract

**Figure:**
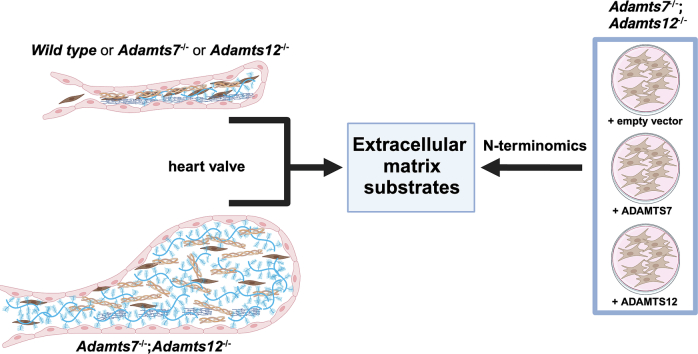

## 1 Introduction

Dysregulated extracellular matrix (ECM) remodeling is implicated in many cardiovascular disorders including atherosclerosis and aortic aneurysms [1,2]. Whereas matrix metalloproteinases (MMPs) are popularly regarded as the principal mediators, there is increasing recognition of a role for ADAMTS proteases, which are a distinct family of nineteen secreted metalloproteases [3]. Several ADAMTS proteases have established roles relevant to the cardiovascular system, i.e., in cardiac development, thrombosis, aortic aneurysms, angiogenesis, atherosclerosis/coronary artery disease and vascular restenosis [3]. Among them, ADAMTS7 and ADAMTS12 are homologous proteases that are unusual in also being chondroitin sulfate proteoglycans [4]. An *ADAMTS7* sequence variant was implicated in increased susceptibility to coronary artery disease and shown to act by promoting smooth muscle cell motility [5]. Manipulation of ADAMTS7 activity in the vascular wall affects smooth muscle proliferation and migration in animal models of vascular injury and disease, and an ADAMTS7 peptide vaccine showed therapeutic benefit in a mouse model of atherosclerosis [6]. Together, ADAMTS7 and ADAMTS12 are known to have a protective role against soft tissue ossification in the musculoskeletal system and *Adamts7*^−/−^;*Adamts12*^−/−^ mice also demonstrated abnormal tendon collagen fibrils, although skeletal development was unaffected [4].

Motivated by the significance of ADAMTS7 in atherosclerosis, where it is now a drug target, and observation of overlapping expression in developing and adult heart valves, we investigated the role of ADAMTS7 and ADAMTS12 in cardiac valve ECM. To uncover and compare their proteolytic activities, we used a specialized proteomics approach, Terminal Amine Isotopic Labeling of Substrates (TAILS), which was previously applied to define ADAMTS7 substrates in aortic endothelial and smooth muscle cell cultures [7], but not fibroblastic ECM. The present investigation reveals a previously unsuspected role of ADAMTS7 and ADAMTS12 in proteostasis of the heart valve ECM and underlying primary mechanisms through identification of their substrates. These insights are potentially valuable for therapeutic targeting of ADAMTS7 [6,8].

## 2 Methods

### 2.1 Transgenic mice

*Adamts7*^*lacZ*^ and *Adamts12* knockout mice (hereafter, homozygous mutants are referred to as *Adamts7*^−/−^and *Adamts12*^−/−^) were previously described [4,9] and were in the C57BL/6 background. *Adamts7*^−/−^*;Adamts12*^−/−^ mice were generated by crossing *Adamts7*^−/−^ mice with *Adamts12*^−/−^ mice. Age-matched wild type C57BL/6 embryos and mice were used as controls for *Adamts7*^−/−^*;Adamts12*^−/−^ mice. Mouse embryos were generated by controlled matings, using the observation of a vaginal mucus plug to designate embryo (E) day 0.5 (E0.5). These studies were approved by the Cleveland Clinic IACUC (protocol no. 00002761).

### 2.2 Echocardiography

As previously described [10], cardiac function (aortic peak velocity, aortic valve peak pressure gradient, fractional shortening and ejection fraction) was assessed in vivo by echocardiography of age-matched wild type and *Adamts7*^−/−^;*Adamts12*^−/−^ mice anesthetized with isoflurane gas (1–2 % with 2 lpm oxygen) to maintain a heart rate between 400 and 500 beats per minute using a GE Vivid7 Ultrasound system equipped with a 14 MHz iL13 probe. To assess fractional shortening and ejection fraction, two-dimensional and M-mode echocardiographic measurements were recorded in the parasternal short axis view of the left ventricle at the level of the papillary muscles. Modified long-axis views were recorded to obtain transaortic valve Doppler flow measurements. Functional parameters were calculated using standard formulas [11].

### 2.3 Real-time quantitative PCR (qRT-PCR)

qRT-PCR was performed as previously described [4]. Briefly, total RNA was isolated using TRIzol (15596018, Invitrogen), and 1 μg of RNA was reverse transcribed into cDNA with SuperScript III Cellsdirect cDNA synthesis system (46-6321, Invitrogen). Power SYBR Green Mastermix (4367659, Applied Biosystems) was used for qRT-PCR using an Applied Biosystems 7500 instrument. *Gapdh* served as the housekeeping gene. The ∆∆Ct method was used to calculate relative mRNA expression levels of target genes and graphed with the use of GraphPad Prism. See Table S1 for primer sequences.

### 2.4 Histology, immunofluorescence and RNA in situ hybridization

All dissected tissues were fixed in freshly prepared 4 % paraformaldehyde (PFA) in phosphate-buffered saline (PBS) at 4 °C overnight followed by paraffin embedding. 7 μm-thick sections on SuperFrost Plus glass slides (Thermo Fisher Scientific) were used for H&E staining, Masson trichrome stain (AB150686; Abcam), modified RGB trichrome stain [12], picrosirius red (26357–02; Electron Microscopy Sciences), Alcian Blue 8GX (A3157; Sigma Aldrich), Alizarin Red S (A5533; Sigma Aldrich) and Movat pentachrome (KTRMPPT; StatLab Medical Products). To quantify valve thickness, the widest portion of the cusps was measured using Fiji software [13]. Valve length, cross-sectional area and cell number were obtained using ImageJ. Three independent measurements from non-consecutive sections were taken per cusp per mouse.

For immunofluorescence staining (antibodies are listed in Table S2), antigen retrieval was used, i.e., immersion of slides in citrate-EDTA buffer (10 mM/L citric acid, 2 mM/L EDTA, 0.05 % v/v Tween-20, pH 6.2) and microwaving for 1.5 min at 50 % power four times in a microwave oven with 30 s intervals intervening prior to antibody incubation. Chondroitinase ABC (0.05 U/μl, diluted 1:200 in 5 % goat serum in PBS; 3667, Sigma-Aldrich) digestion of sections was used prior to aggrecan immunostaining [14]. β-galactosidase staining of hearts from *Adamts7*^lacZ/+^ mice was performed as previously described, followed by paraffin embedding and counterstaining of 7 μm sections with eosin for microscopy [4,15]. Apoptosis was measured using a commercial TUNEL assay (11684795910 Roche Diagnostics). *Adamts12* RNA in situ hybridization was performed using RNAScope (Advanced Cell Diagnostics) as previously described [4,16]. Briefly, 6 μm sections were deparaffinized and hybridized to a mouse *Adamts12* probe set (400531; Advanced Cell Diagnostics) using a HybEZ™ oven (Advanced Cell Diagnostics) and the RNAScope 2.5 HD Detection Reagent Kit (322,360; Advanced Cell Diagnostics).

### 2.5 Synchrotron imaging

4 % PFA-fixed, paraffin-embedded hearts were imaged with synchrotron-based phase-contrast micro-computed tomography at the TOMCAT beamline of the Swiss Light Source (Paul Scherrer Institut, Villigen, Switzerland) applying previously described experimental settings [17]. Phase-contrast was achieved with free space propagation. The x-ray beam was monochromatized to an energy of 21 keV. The sample was placed on a rotating stage in-between source and detector, with a sample-to-detector distance of 19 cm to get optimal propagation. The detecting system, consisting of a 20 μm thick LuAG:Ce scintillator, a 4× magnifying objective and a sCMOS detector, resulted in a 4.2 × 3.5 mm^2^ field-of-view with an effective pixel size of 1.63 × 1.63 μm^2^. A full tomographic scan consisted of a 180° rotation of the sample, during which 1801 projection images were acquired at regular intervals. Each projection had an acquisition time of 80 ms, resulting in a total scan time of about 2.4 min per sample. Following acquisition, phase-retrieval was performed using Paganin's method (δ = 3.7 ∗ 10^−8^, β = 1.7 ∗ 10^−10^) [18]. The volumes were then reconstructed from the projections by using the gridrec algorithm [18]. The obtained data were volumes of 2560 × 2560 × 2160 pixels, with a 16-bit pixel depth. The data were then visualized with a combination of the softwares Fiji [13] and Amira (Thermo Fisher Scientific).

### 2.6 Isolation of Adamts7^−/−^;Adamts12^−/−^ and wild type mouse embryo fibroblast (MEFs) matrisomes

*Adamts7*^−/−^;*Adamts12*^−/−^ and wild type MEFs (n = 3 each) were cultured to confluence in 10 cm^2^ plates as described above and incubated with indicator-free, serum-free medium for 24 h and the medium was collected and centrifuged at 1500 ×*g* for 5 min to minimize cellular debris. The cell monolayer was trypsinized gently by incubation with a thin film of trypsin-EDTA (25,200,056; ThermoFisher Scientific) for 3 min. 1 ml of serum free medium was added, pipetted gently to detach the cells and the cell suspension was centrifuged at 1500 ×*g* for 5 min. The cell pellet was discarded and supernatant was collected and added to the centrifuged medium. This sample, comprising the secretome and matrisome, was subjected to buffer exchange and transferred to 50 mM ammonium bicarbonate (ABC) using 3 kDa molecular weight cutoff (MWCO) filters. Total protein was measured using the Bradford assay and the protein concentration was adjusted to 1 mg/ml with 50 mM ABC. 50 μg of total protein from each sample was taken for proteomics. Protein was reduced with 5 mM 1,4-dithiothreitol (DTT, final concentration) and incubated at 60 °C for 1 h and alkylated with 15 mM iodoacetamide (IAA) final in the dark for 20 min at room temperature, and the reaction was quenched by adding 10 mM final concentration of DTT at 37 °C for 30 min. Trypsin Gold (V5280; Promega) was added in 1:100 (trypsin to protein) ratio for overnight digestion at 37 °C. Samples were cleaned using C18 columns (WAT054955; Waters, Sep-Pak). Final eluate was collected and dried and the peptides were reconstituted in 1 % acetic acid.

### 2.7 Sample preparation for TAILS of ADAMTS7 and ADAMTS12 substrates

E14.5 *Adamts7*^*−/−*^*;Adamts12*^−/−^ MEFs were cultured in 10 cm^2^ plastic culture dishes in Dulbecco's Modified Eagle Medium (DMEM) supplemented with 10 % fetal bovine serum (FBS), 100 U/ml penicillin, and 100 μg/ml streptomycin at 37 °C in a 5 % CO_2_ humidified chamber. HEK293F cells were transiently transfected with control or *Adamts7* or *ADAMTS12* expression plasmids (from S. Cal, University of Oviedo, Oviedo, Spain) using PEI MAX (24,765; Polysciences) and co-cultured with the MEFs in a 80 (MEF):20 (HEK) ratio. Empty vector transfected HEK293F cells were used for co-culture in the control experiment. 18 h post transfection, the cells were given 10 ml indicator-free, serum-free medium which was collected 24 h later. Protease inhibitor cocktail (11,836,153,001; Millipore Sigma) was added to the medium to achieve 1× concentration followed by concentration using a Speedvac. After methanol chloroform precipitation, protein pellets were resolubilized in the minimum volume of 6 M GuHCl and samples were transferred to 100 mM HEPES pH 7.5 by buffer exchange using 3 kDa MWCO filters. Total protein was determined using Bradford assay. 200 μg of protein per sample was transferred to a fresh tube, reduced using 5 mM DTT (final concentration) and incubation at 60 °C for 1 h and samples were alkylated using 15 mM IAA final by incubating for 30 min in the dark at room temperature. The reaction was stopped using 10 mM DTT (final concentration) and incubation for 30 min at 37 °C.

### 2.8 Labeling and enrichment of protein N-termini for TAILS

Proteins from the culture medium of active ADAMTS7/ADAMTS12-exposed and control cultures (n = 3 each) were labeled by adding 40 mM final concentration of heavy (596,388; Millipore Sigma) and light formaldehyde (ULM-9498-100; Cambridge Isotope Laboratories, Inc), respectively, followed immediately by addition of sodium cyanoborohydride at 20 mM final concentration in the fume hood, pH was adjusted between 6 and 7 and incubated at 37 °C for 16 h. Fresh formaldehyde (20 mM final), along with 10 mM final sodium cyanoborohydride were added and further incubated for 3 h. The reaction was quenched by adding 100 mM (final) Tris pH 6.8 and incubation at 37 °C for 1 h. pH was adjusted to 8 using 1 M HEPES. Paired heavy and light-labeled samples were mixed and digested overnight with trypsin at a 1:100 (trypsin:protein) ratio. A small volume containing approximately 20 μg of peptides was retained as a preTAILS sample for assessment of labeling and enrichment efficiency.

For multiplexed TAILS, 8-plex iTRAQ isobars (4381664; SCIEX) were reconstituted using dimethyl sulfoxide (DMSO) to a final concentration of 50 %. Each sample was incubated with 25 μg of the isobars for 2 h in the dark. The reaction was quenched by adding 100 mM ammonium bicarbonate and the samples were combined, pH adjusted to 8 using 1 M HEPES and digested with trypsin overnight at a 1:100 (trypsin: protein) ratio. A small volume containing approximately 20 μg of peptides was retained (preTAILS sample).

The peptides from dimethyl and iTRAQ labeling were incubated with hyperbranched polyglycerol-aldehydes (HPG-ALD, Flintbox, https://www.flintbox.com/public/project/1948/) at a 5:1 polymer: protein ratio. The mixtures were filtered through 10 kDa MWCO filters (EMD Millipore Corp.) and the unbound peptides comprising non-reactive (N-terminally labeled and blocked) peptides were collected as the flow-through. These peptides comprise TAILS samples. Pre-TAILS and TAILS samples were desalted on a Sep-Pac (Waters). Samples were vacuum centrifuged until dry and re-suspended in 1 % acetic acid.

### 2.9 Mass spectrometry

Peptides were analyzed on a Fusion Lumos tribrid mass spectrometer system (ThermoFisher Scientific) interfaced with a Thermo Ultimate 3000 nano-UHPLC. The HPLC column was a Dionex 15 cm × 75 μm id Acclaim Pepmap C18, 2 μm, 100 Å reversed phase capillary chromatography column. 5 μL volumes of the trypsin-digested extract were injected, peptides were eluted from the column by an acetonitrile/0.1 % formic acid gradient at a flow rate of 0.3 μL/min and introduced in-line into the mass spectrometer over a 120 min gradient. The nanospray ion source was operated at 1.9 kV. The digest was analyzed using a data-dependent method with 35 % collision-induced dissociation fragmentation of the most abundant peptides every 3 s and an isolation window of 0.7 *m*/*z* for ion-trap MS/MS. Scans were conducted at a maximum resolution of 120,000 for full MS. Dynamic exclusion was enabled with a repeat count of 1 and ions within 10 ppm of the fragmented mass were excluded for 60 s.

### 2.10 Proteomics and TAILS data analysis

For matrisome shotgun analysis, .raw files were imported into Proteome Discoverer 2.3 (PD 2.3) (Thermo Fisher Scientific). Analysis parameters were set as precursor mass tolerance of 10 ppm, and fragment mass tolerance of 0.6 Da. Static modification was set as carbamidomethyl (C), whereas dynamic modifications included oxidation (M). Spectra were searched using mouse database (downloaded on 06/29/2022 with 25,261 protein sequences) and human database (downloaded on 11/27/2022 with 42,357 protein sequences) from UniProt. False discovery rate (FDR) was calculated by creating a decoy database using percolator node from PD 2.3 and a strict cutoff was applied at 1 % FDR. The presence of two or more high confidence peptides designated a protein.

For TAILS data analysis mass spectrometer .raw files were imported into PD 2.3 (Thermo Fisher Scientific). Analysis parameters were set as precursor mass tolerance of 10 ppm, and fragment mass tolerance of 0.6 Da. Static modification was set as carbamidomethyl (C), whereas dynamic modifications included the light (+28.031 Da) dimethyl formaldehyde (N-terminal, K), Heavy (+34.063) dimethyl formaldehyde (N-terminal, K) or 8plex iTRAQ (N-terminal, K), oxidation (M, P), deamidation (N, R), acetylation (N-terminal), and Gln to pyro-Glu N-terminal.

### 2.11 Positional peptide mapping, statistics and identification of proteolytically cleaved peptides

Peptide groups from PD2.3 were exported as an Excel file for pre-TAILS and TAILS samples and combined. Peptides modified at the N-terminus and with quantitative values were retained and positionally mapped on the protein sequence using TopFind 4.0 (https://topfind.clip.msl.ubc.ca/topfinder). Positionally internal N-termini (i.e., not peptides with initiator methionine present or removed, N-termini generated by signal peptide/propeptide/transit peptide removal) were considered as potential proteolytically cleaved peptides. All proteolytic peptides from TAILS analysis were imported into Perseus 1.6.5.0 (https://maxquant.net/perseus/), intensity values were log transformed, peptides were filtered out based on valid values in at least 70 % samples, missing values were imputed from the normal distribution (width = 0.3 and down shift = 1.8), Student *t*-test was applied with a Benjamini-Hochberg FDR correction to determine significantly enriched peptides in the active enzyme group with p < 0.05.

### 2.12 Statistical methods

**Fig. 1:**
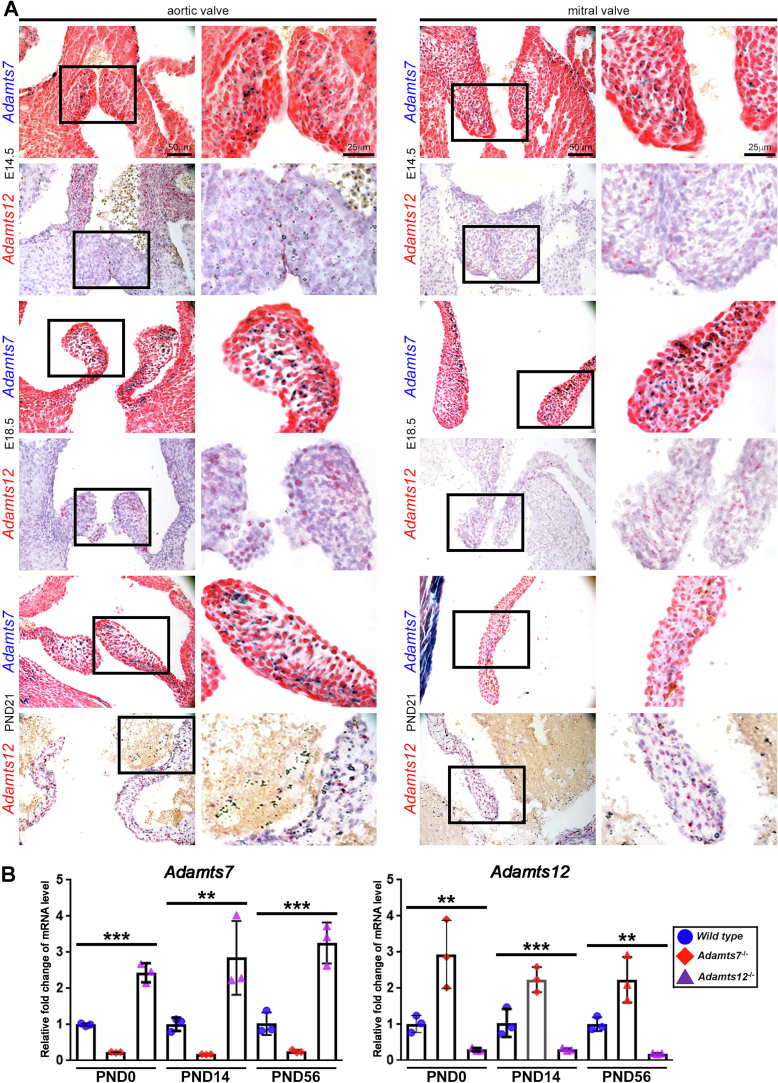
*Adamts7* and *Adamts12* are coordinately expressed in aortic and mitral valves and show compensatory upregulation in gene knockouts. **A**. *Adamts7* and *Adamts12* expression is revealed in embryonic and 3-week-old mouse hearts by LacZ (blue) and RNA in situ hybridization (red), respectively. Both genes are expressed in aortic and mitral valves during development and maturation. n=4 biological replicates and two technical replicates for each time point and stain. **B**. qRT-PCR analysis of *Adamts7* and *Adamts12* mRNAs in wild type, *Adamts7*^−/−^ and *Adamts12*^−/−^ mice shows compensatory upregulation of each in the absence of the other. N = 3 biological replicates and three technical replicates. One way ANOVA was used for statistical analysis. Error bars represent SEM. *p ≤ 0.05, **p ≤ 0.01, ***p ≤ 0.001. E, embryonic day; PND, postnatal day. (For interpretation of the references to color in this figure legend, the reader is referred to the web version of this article.)

**Fig. 2:**
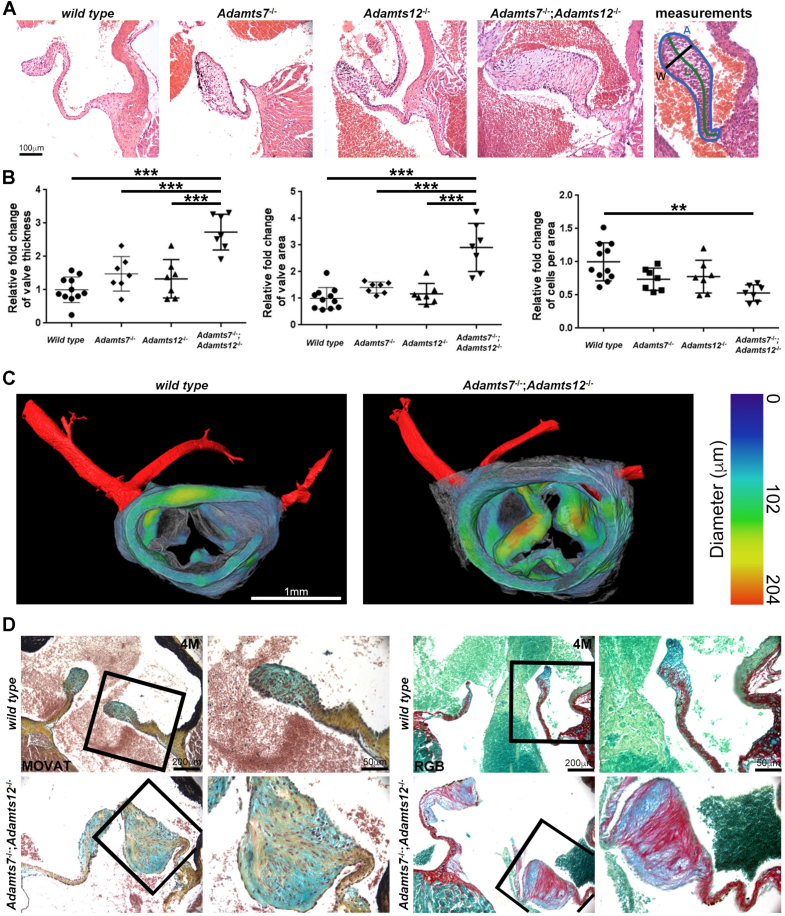
*Adamts7*^−/−^;*Adamts12*^−/−^ aortic valves are enlarged and disorganized postnatally. **A–B**. Hematoxylin and eosin stained two-month (2 M) and four-month-old (4 M) aortic valves from *Adamts7*^−/−^;*Adamts12*^−/−^ aortic valves (**A**) have increased thickness and area with reduced cell number, as compared to control (**B**). Note *Adamts7*^−/−^ aortic valves have increased area and decreased cell density as compared to control (**B**). n = 11 wild type, 7 *Adamts7*^−/−^, 7 *Adamts12*^−/−^, 7 *Adamts7*^−/−^;*Adamts12*^−/−^. Duplicate analysis was performed. Error bars represent SEM. One-way ANOVA followed by Tukey test was used for statistical analysis. *p ≤ 0.05, **p ≤ 0.01, ***p ≤ 0.001. **C.** Synchrotron imaging reveals thickened aortic valve leaflets as compared to control. The images are color coded to illustrate the relative valve thickness. Coronary arteries are shown in red at the top of the aortic root. **D**. Four-month-old histological sections of *Adamts7*^−/−^;*Adamts12*^−/−^ aortic valves show disorganized collagen distribution (yellow staining, left panels; Picrosirius red stain (red), right panels) and proteoglycan staining (Movat Pentachrome stain (blue, left panels); alcian blue staining (blue), right panels) and loss of normal elastin content (black, left panels) as compared to control. N = 4 aortic valves for each genotype. (For interpretation of the references to color in this figure legend, the reader is referred to the web version of this article.)

Statistical analysis was performed using Prism 10.3.0. Data from this study are presented as the standard error of the mean. One-way ANOVA (Fig. 1B), one-way ANOVA followed by Tukey test (Fig. 2B) and unpaired Student *t*-tests (all other quantified data) were performed to assess statistical significance. *p ≤ 0.05, **p ≤ 0.01, *** p ≤ 0.001.

## 3 Results

### 3.1 *Adamts7* and *Adamts12* are co- expressed in murine heart valves

**Fig. 3:**
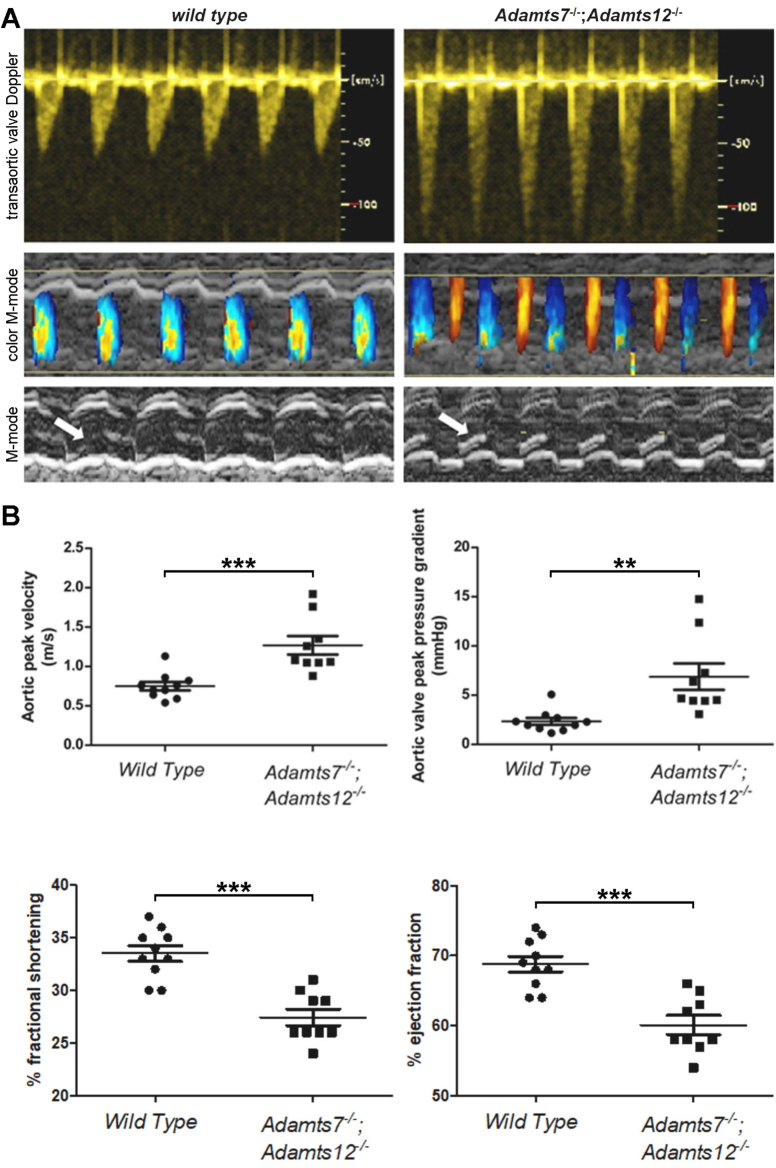
Assessment of systolic flow through pulse wave-Doppler analysis. **A**. Representative transaortic valve Doppler images (top panels) and color M-mode ultrasound (middle panels). Note regurgitant (red colored) flow in *Adamts7*^−/−^;*Adamts12*^−/−^ mice as compared to control (middle panels). Representative two-dimensional M-mode ultrasound images of control and *Adamts7*^−/−^;*Adamts12*^−/−^ aortic valves (bottom panels) illustrating thicker *Adamts7*^−/−^;*Adamts12*^−/−^ valves (white arrows). **B**. Aortic peak velocity and pressure gradient (top panels) are increased in *Adamts7*^−/−^;*Adamts12*^−/−^ mice. Assessment of cardiac systolic function in the left ventricle of wild type and *Adamts7*^−/−^;*Adamts12*^−/−^ mice shows reduced percent ejection fraction and percent fractional shortening in *Adamts7*^−/−^;*Adamts12*^−/−^ mice (bottom panels) as compared to control. n = 10 wild type, 9 *Adamts7*^−/−^;*Adamts12*^−/−^. Each measurement was performed in duplicate. Error bars represent SEM. An unpaired Student *t*-test was used for statistical analysis. *p ≤ 0.05, **p ≤ 0.01, ***p ≤ 0.001.

Analysis of *Adamts7* mRNA expression by β-galactosidase staining in *Adamts7*^LacZ/+^ hearts and of *Adamts12* using RNA in situ hybridization (ISH) in wild type hearts demonstrated expression of each gene in embryonic valve cushion mesenchyme and valvular interstitial cells, but not the endocardium of embryonic and postnatal 21-day old valves, respectively (Fig. 1A, Fig. S1A–B). Expression was noted throughout the ventricles and atria (Fig. S1C–D), but it was not possible to specify a cell type. However, scRNA sequencing of wild type 6-day old mouse hearts previously showed scattered *Adamts7* expression in cardiomyocyte, fibroblast and endothelial clusters, whereas *Adamts12* was preferentially expressed in cardiac fibroblasts and pericytes/smooth muscle cells [19]. *Adamts12* but not *Adamts7*, was expressed in the aortic tunica media (Fig. 1A). In the human heart, *ADAMTS7* was previously shown to be expressed by atrial and ventricular cardiomyocytes, smooth muscle cells and fibroblasts, and *ADAMTS12* was also widely expressed, mostly in atrial cardiomyocytes and pericytes [20]. qRT-PCR analysis of *Adamts7* mRNA in *Adamts12*^−/−^ hearts identified its upregulation after *Adamts12* inactivation and *Adamts12* mRNA was upregulated in *Adamts7*^−/−^ hearts (Fig. 1B), implying that these homologous genes are susceptible to the recently described phenomenon of transcriptional adaptation [21,22], known to affect their expression in limb development [4]. Hearts from the single gene mutants were morphologically and histologically normal. To uncover roles that may be potentially masked in single mutants by transcriptional adaptation and reveal cooperative functions resulting from spatially and temporally overlapping expression, *Adamts7*^−/−^;*Adamts12*^−/−^ mice were generated by interbreeding the respective mutants. As previously described, the double knockout mice were externally unremarkable, with normal life expectancy [4] and with comparable heart weight/body weight and heart weight/tibial length ratios as controls (Fig. S2). However, 4-month-old *Adamts7*^−/−^;*Adamts12*^−/−^ aortic valve leaflets had increased pigmentation and a significant increase in width and area with an accompanying reduction in cell density as compared to wild type and single mutant mice (Fig. 2A, B, Fig. S3). Synchrotron imaging confirmed greater valve leaflet thickness in *Adamts7*^−/−^;*Adamts12*^−/−^ hearts (Fig. 2C). Proteoglycan staining using Movat pentachrome, RGB and Alcian blue stains, was increased in *Adamts7*^−/−^;*Adamts12*^−/−^ aortic valve leaflets (Fig. 2D, Fig. S4). Collagen staining by RGB and picrosirius red stains was also more intense and collagen fibers appeared disorganized in 4-month-old adult leaflets (Fig. 2D, Fig. S4). Elastin distribution was disrupted in the ventricularis layer of *Adamts7*^−/−^;*Adamts12*^−/−^ aortic valve leaflets (Fig. S4). Valve calcification, assessed using alizarin red staining, was absent in control and *Adamts7*^−/−^;*Adamts12*^−/−^ mice (Fig. S5). *Adamts7*^−/−^;*Adamts12*^−/−^ pulmonic, mitral and tricuspid valve leaflets were also enlarged relative to controls (Fig. S6). Consistent with aortic valve dysmorphology, echocardiographic analysis of 4-month-old *Adamts7*^−/−^;*Adamts12*^−/−^ mice revealed aortic stenosis and regurgitation, reduced fractional shortening and reduced left ventricular ejection fraction. Peak flow velocity across the aortic valve increased from an average of 0.7 m/s for wild type mice to 1.3 m/s in *Adamts7*^−/−^;*Adamts12*^−/−^ mice with significantly increased systolic pressure gradients (Fig. 3A, B). Two-dimensional M-mode echocardiography also revealed depressed left ventricular ejection fraction (LVEF) and fractional shortening (FS) and increased diastolic and systolic LV chamber dimensions in *Adamts7*^−/−^;*Adamts12*^−/−^ mice (Fig. 3B). These findings indicate valvular dysfunction, presenting hemodynamic features of both aortic stenosis and regurgitation. The prevalence of altered hemodynamics, as defined by color Doppler imaging of turbulent blood flow through the aortic valve, was 30 % (3/10 mice) at 4 months of age and 60 % (9/15 mice) at 12 months of age, in contrast to normal flow in wild type littermates (Table S3).

### 3.2 ECM dysregulation in *Adamts7*^−/−^;*Adamts12*^−/−^ aortic valve leaflets

**Fig. 4:**
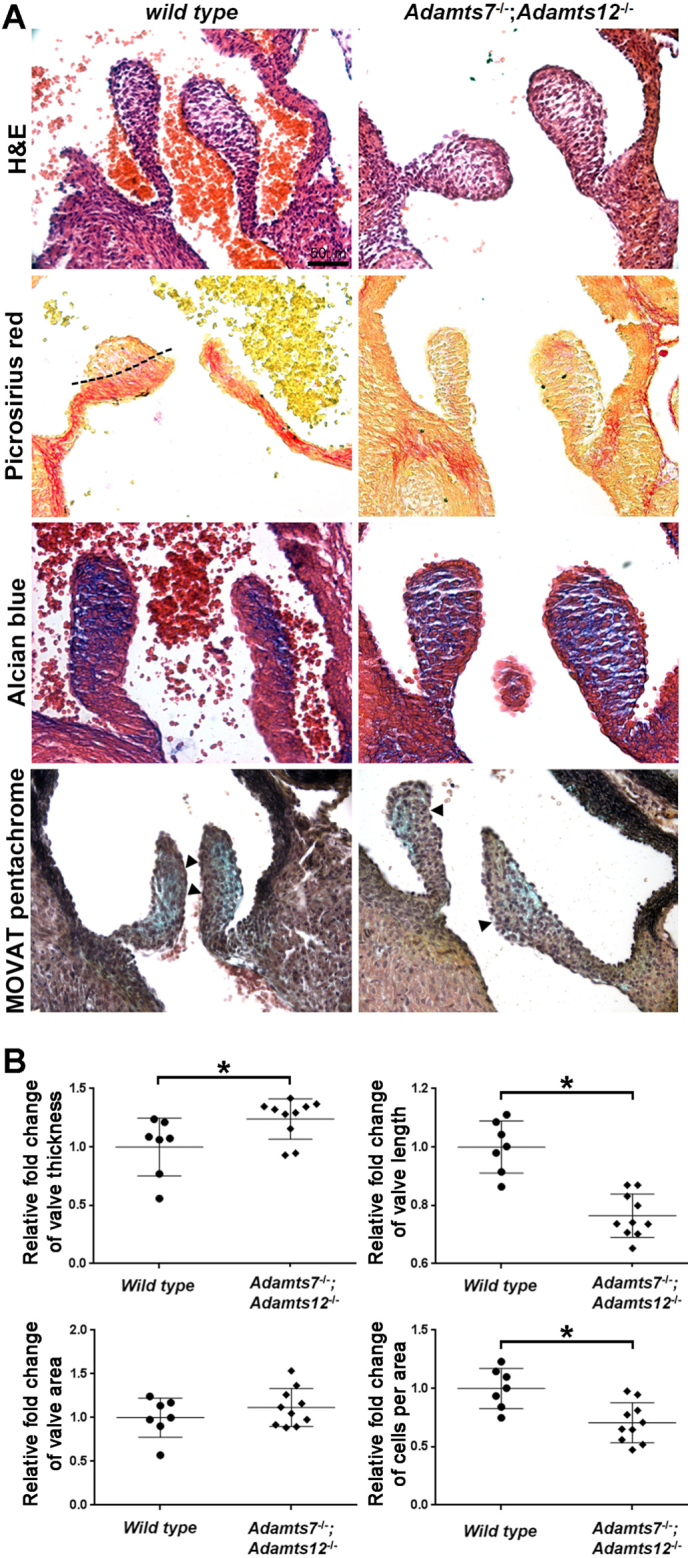
Enlargement of *Adamts7*^−/−^;*Adamts12*^−/−^ aortic valves is initiated during development. **A**. E18.5 *Adamts7*^−/−^;*Adamts12*^−/−^ aortic valves are enlarged, as assessed by hematoxylin and eosin-stained sections, have disorganized collagen (picrosirius red), increased proteoglycans (alcian blue and Movat pentachrome). The dashed line demarcates collagen. Arrowheads show the elastic fibers in wild type valves and lack of elastin in *Adamts7*^−/−^;*Adamts12*^−/−^ aortic valves. The analysis used n=4 of each genotype and two technical replicates for each stain. **B**. *Adamts7*^−/−^;*Adamts12*^−/−^ aortic valves have increased thickness and length and reduced cell number, as compared to control. n = 7 wild type, 10 *Adamts7*^−/−^;*Adamts12*^−/−^. Each stain was done on two or more sections from each heart. Error bars represent SEM. An unpaired Student *t*-test was used for statistical analysis. *p ≤ 0.05. (For interpretation of the references to color in this figure legend, the reader is referred to the web version of this article.)

**Fig. 5:**
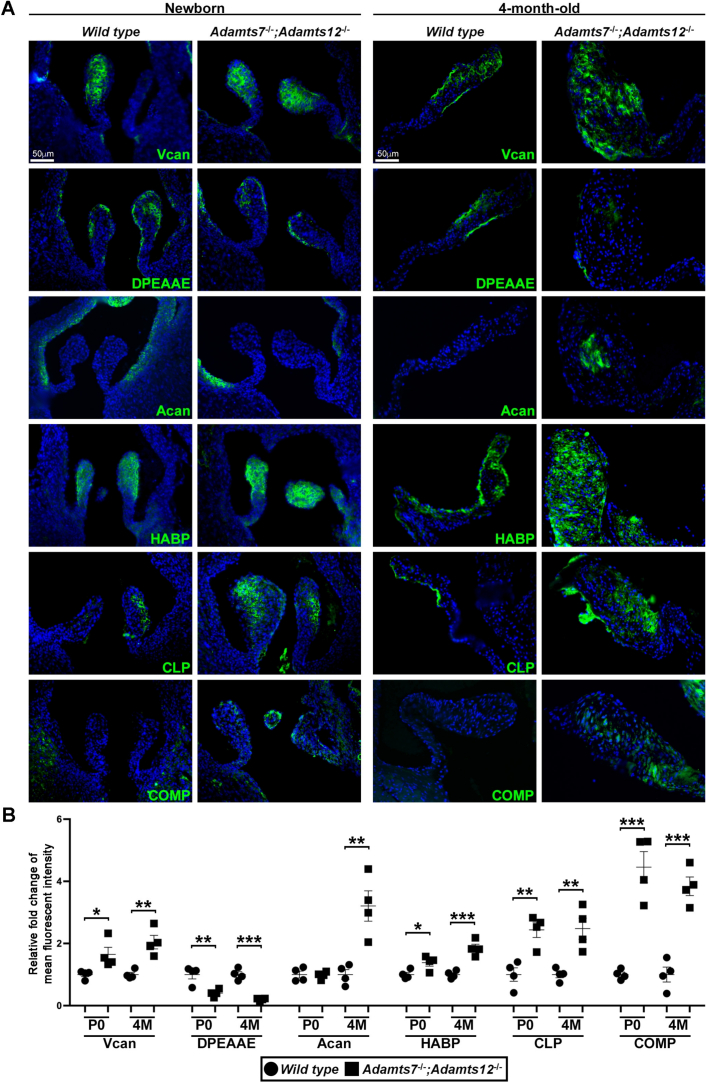
*Adamts7*^−/−^;*Adamts12*^−/−^ aortic valves have increased proteoglycan and glycosaminoglycan staining, with reduced versican proteolysis. Newborn and four-month-old histological sections of *Adamts7*^−/−^;*Adamts12*^−/−^ aortic valves show increased versican staining and reduced staining intensity of cleaved versican (DPEAAE) as compared to littermate controls. HABP and CLP accumulated in *Adamts7*^−/−^;*Adamts12*^−/−^ aortic valves with ectopic COMP in newborn and adult and ectopic aggrecan in adult aortic valves. n=4 mice of each genotype, with two technical replicates for each genotype and time point. Error bars represent SEM. A paired Student t-test was used for statistical analysis. *p ≤ 0.05, **p ≤ 0.01, ***p ≤ 0.001.

To ascertain whether valve anomalies noted first at 4 months had developmental origins, we analyzed valves near completion of gestation (18.5 days), by which time leaflet development is substantially complete. *Adamts7*^−/−^;*Adamts12*^−/−^ aortic valve leaflets were short and stout compared to wild type leaflets (Fig. 4A). Wild type leaflets displayed well-organized ECM lamination, with a tension-side (ventricularis) enrichment of elastin and compression-side enrichment of proteoglycans (Fig. 4A, alcian blue staining) and a clearly demarcated collagen layer (Fig. 4A, dashed line). *Adamts7*^−/−^;*Adamts12*^−/−^ leaflets had more extensive proteoglycan staining, poorly localized collagen, and lacked a distinct compact elastin layer (Fig. 4A, arrowheads), indicating abnormal ECM organization. Consistent with the observed difference in shape, morphometric evaluation showed increased leaflet thickness, reduced leaflet length and lower cell density (Fig. 4B). Due to greater proteoglycan staining in the *Adamts7*^−/−^;*Adamts12*^−/−^ aortic valve leaflets, we assessed distribution of versican (VC), an abundant proteoglycan in developing semilunar valves and substrate of several ADAMTS proteases. Staining in newborn *Adamts7*^−/−^;*Adamts12*^−/−^ aortic valve leaflets was comparable to wild type, but increased versican staining was observed in 4-month-old *Adamts7*^−/−^;*Adamts12*^−/−^ aortic valve leaflets (Fig. 5). Conversely, staining for ADAMTS protease-cleaved versican (DP), was considerably reduced in 4-month-old *Adamts7*^−/−^;*Adamts12*^−/−^ aortic valve leaflets (Fig. 5). 4-month-old *Adamts7*^−/−^;*Adamts12*^−/−^ aortic valve leaflets had strong staining for the related proteoglycan aggrecan (Acan) which was absent in wild type valves, and dramatically increased staining intensity with hyaluronan-binding protein (HABP). Versican and aggrecan bind cartilage link protein and HA, forming large hydrophilic aggregates (Fig. 5). Cartilage link protein (CLP) also showed increased staining in *Adamts7*^−/−^;*Adamts12*^−/−^ aortic valve leaflets. In addition, staining of a previously identified substrate, COMP, was observed in *Adamts7*^−/−^;*Adamts12*^−/−^ aortic valve leaflets, but not in control valves (Fig. 5). In contrast to their tendons [4], there was no change in distribution or staining intensity of small leucine-rich proteoglycans biglycan, fibromodulin or decorin within *Adamts7*^−/−^;*Adamts12*^−/−^ aortic valve leaflets (Fig. S7). There was no accompanying change in proliferation or cell death in *Adamts7*^−/−^;*Adamts12*^−/−^ aortic valves as compared to control (Fig. S8). Together, the leaflet thickening, disorganized collagen and elastin distribution and excess proteoglycan throughout the leaflet were consistent with myxomatous degeneration. Furthermore, nodules of cartilage were observed in the vicinity of the annulus of some (4/19) *Adamts7*^−/−^;*Adamts12*^−/−^ pulmonic valves (Fig. S9).

### 3.3 Identification of ADAMTS7 and ADAMTS12 substrates

Until recently, only a handful of substrates were reported for ADAMTS7 or ADAMTS12 including COMP, CTGF, and TSP1 [[23], [24], [25]]. Using the mass-spectrometry based N-terminomics approach, iTRAQ-TAILS, Colige et al. identified several additional ADAMTS7 substrates using cultured human skin fibroblasts as the source of an ECM substrate library [26]. Because of the observed cooperative effect of ADAMTS7 and ADAMTS12 inactivation on heart valve proteostasis, for which one potential explanation is an overlapping substrate profile, we used TAILS to compare and contrast the ADAMTS7 and ADAMTS12 substrate and cleavage site profiles. We used E14.5 mouse embryonic fibroblasts (MEFs) isolated from *Adamts7*^−/−^;*Adamts12*^−/−^ mice to provide a library of endogenous, soluble and assembled ECM for digestion by exogenous ADAMTS7 and ADAMTS12. The lack of ADAMTS7 and ADAMTS12 in the MEFs was intended to increase the yield of the TAILS experiment in the absence of background proteolysis by ADAMTS7 and ADAMTS12.

**Fig. 6:**
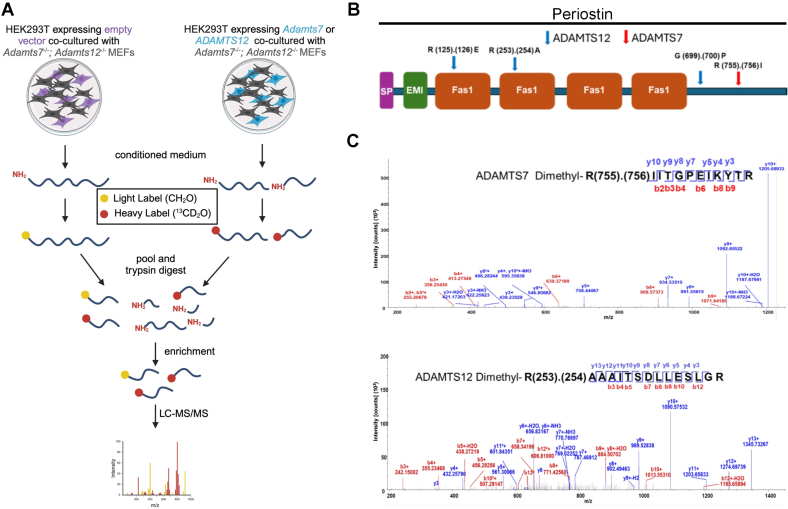
Identification of ADAMTS7 and ADAMTS12 substrates. **A.** Conditioned medium collected from *Adamts7*^−/−^;*Adamts12*^−/−^ mouse embryonic fibroblasts transfected with ADAMTS7, ADAMTS12 or empty vector and TAILS workflow showing sample generation and subsequent handling. **B.** Cleavage sites of periostin by ADAMTS7 and ADAMTS12. **C.** Annotated MS^2^ spectrum examples of ADAMTS7 and ADAMTS12-generated dimethyl peptides, showing b-(N-terminus preserved) and y-type (C-terminus preserved) ions generated by amide bond cleavage during collisional-induced dissociation.

We first undertook a detailed proteomics analysis of mouse embryo fibroblasts to determine whether their matrisome resembled that of normal mouse heart valves [27]. The analysis identified substantial overlap between matrisomes of wild type and *Adamts7*^−/−^;*Adamts12*^−/−^ fibroblasts and mouse valve demonstrating that the MEF substrate library was appropriate for mechanistic investigation of the observed valve defects (Fig. 6A). Since Colige et al. had earlier identified ADAMTS7 candidate substrates using iTRAQ-TAILS [26], we used iTRAQ TAILS as well as dimethyl-TAILS to define the ADAMTS12 degradome, which was previously unknown. To achieve a better comparison of the ADAMTS7 and ADAMTS12 degradomes using identical experimental approaches, we therefore also undertook dimethyl-TAILS seeking ADAMTS7 substrates.

Using TAILS, we identified proteolytically cleaved peptides in a MEF matrisome substrate library digested with ADAMTS7 and ADAMTS12 (Tables S2, S3). Because the medium from the co-cultures used in our TAILS approach contained mouse proteins produced by MEFs as well as human proteins made by HEK293F cells, any of which could be potentially cleaved by ADAMTS7 and ADAMTS12, we searched both mouse and human proteomes to identify all cleavage sites in our ADAMTS7 and ADAMTS12 degradomic analysis. Because ADAMTS7 and ADAMTS12 are secreted proteases, we filtered the data to include only secreted or cell-surface substrates, or lumenal regions of proteins present in the secretory pathway for further consideration (Tables S4, S5, Supplemental Data File).

For ADAMTS7, we identified 55 cleavages deduced from statistically significant N-terminally labeled internal peptides from 32 proteins, with the most numerous cleavages identified in ECM components, especially fibronectin (9 cleavage sites) and collagen I (9 total; 4 and 5 cleavages in the α1 and α2 chains, respectively) (Table S4). The corresponding statistically significant peptides for ADAMTS12 obtained by applying both dimethyl-TAILS and iTRAQ-TAILS identified a total of 94 cleavages in 52 proteins (Table S5), the majority also occurring in ECM components. iTRAQ and dimethyl labeling led to identification of different ADAMTS12 substrates and cleavage sites (see Discussion). 8 of the 52 protein substrates were found using both methods, but only 2 dimethyl or iTRAQ-labeled ADAMTS12-TAILS peptides were identical, one in α-2-HS-glycoprotein and one in gelsolin. The mass spectrometry proteomics data were deposited to the ProteomeXchange Consortium via the PRIDE partner repository with the dataset identifier PXD055550 and Project DOI: https://doi.org/10.6019/PXD055550.

### 3.4 Shared substrates of ADAMTS7 and ADAMTS12

Eleven proteins identified as substrates overlapped between ADAMTS7 and ADAMTS12 (Table 1). However, only three cleavage sites, specifically, in agrin at Lys^1745^-Ser^1746^ (mouse sequence enumeration; the corresponding peptide identified in the ADAMTS12 degradome was a human peptide indicating cleavage at Lys^1863^-Ser^1864^) and in the collagen I alpha-2 chain at Arg^984^-Gly^985^ and Arg^1073^-Ser^1074^ were shared between ADAMTS7 and ADAMTS12 (Table 1).

**Table 1:**
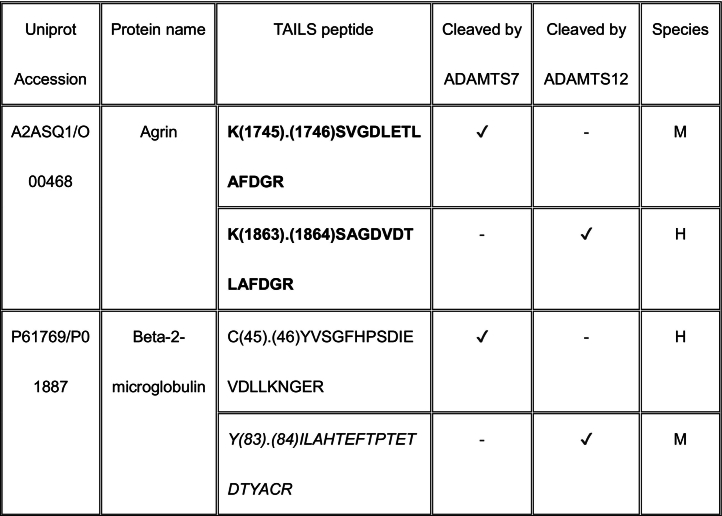

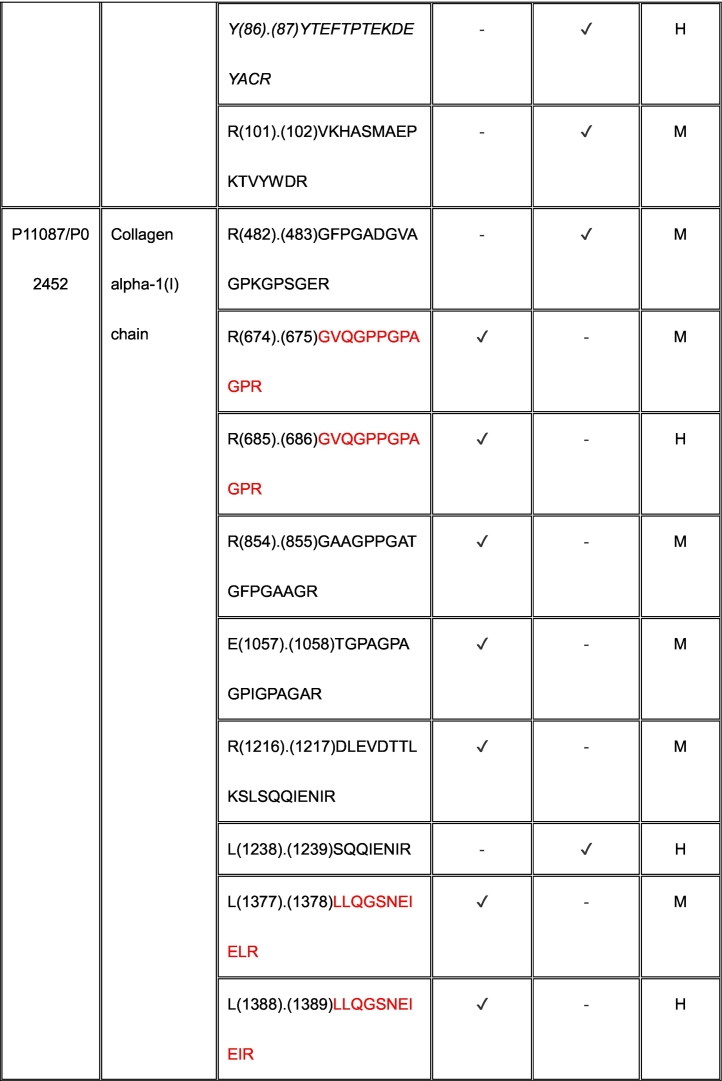

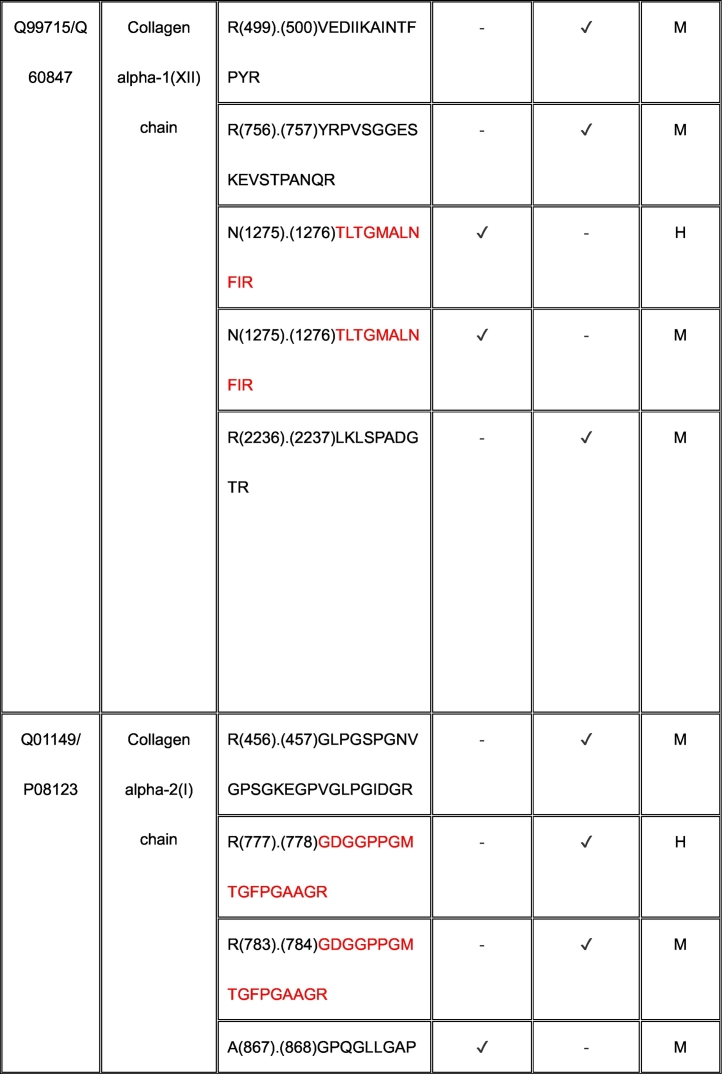

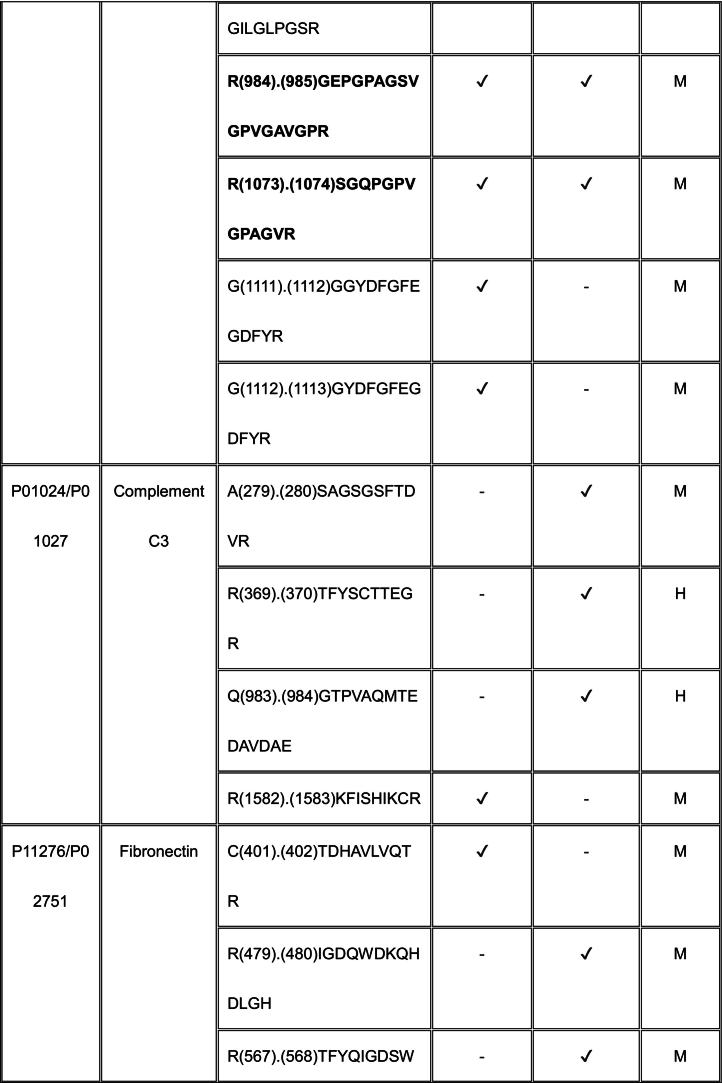

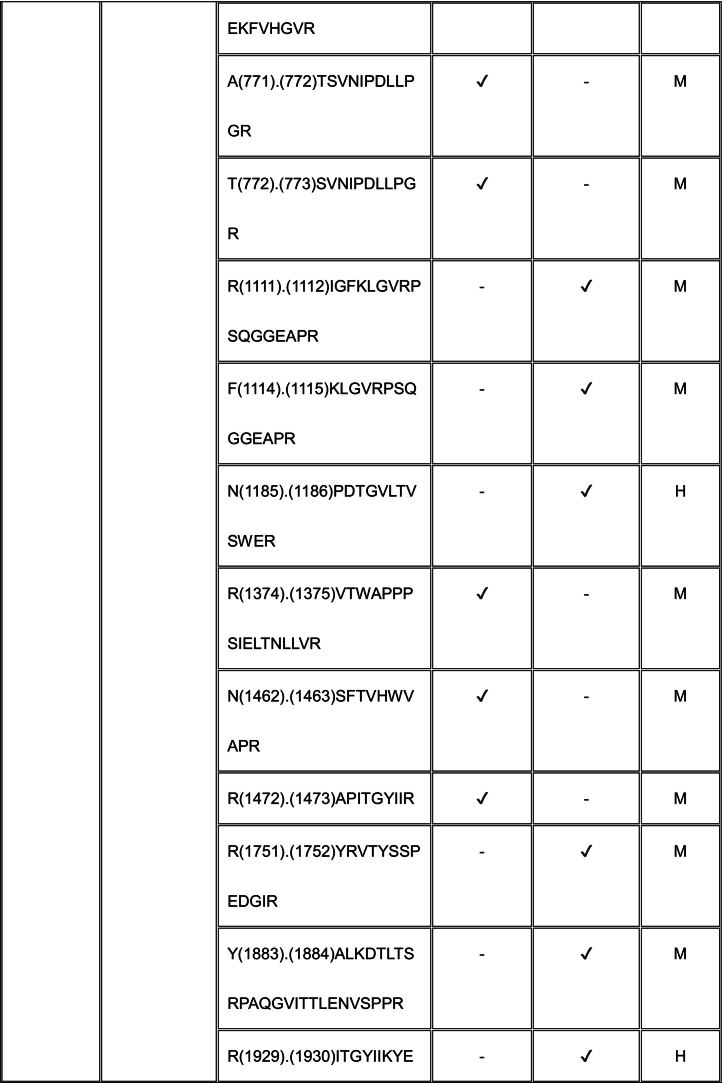

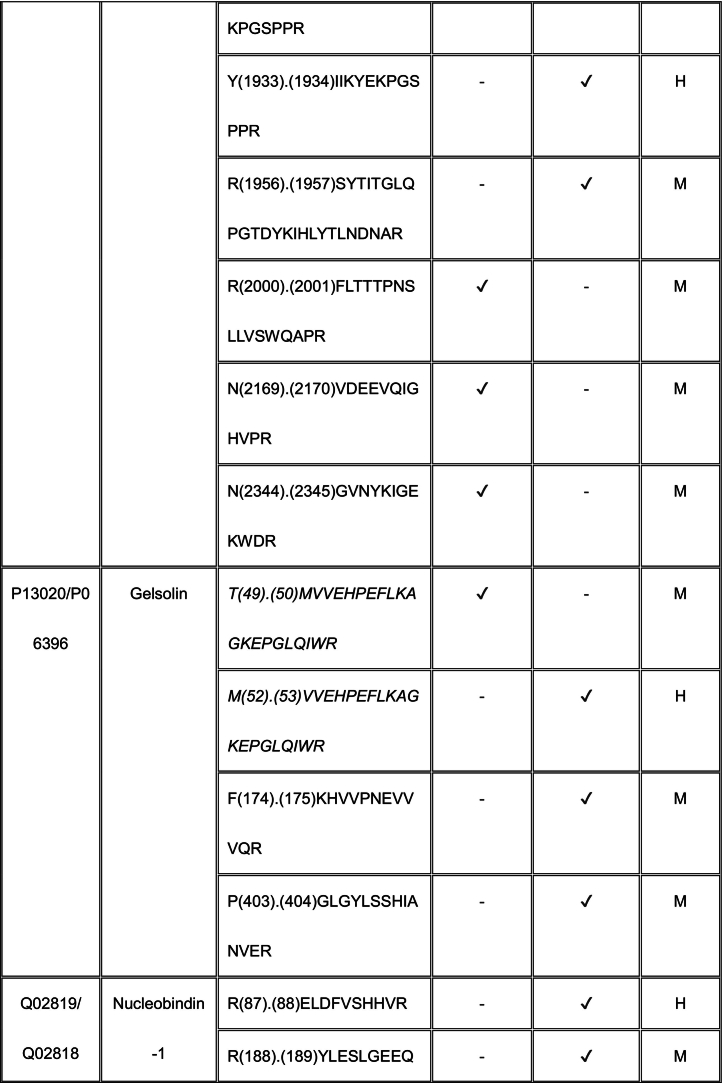

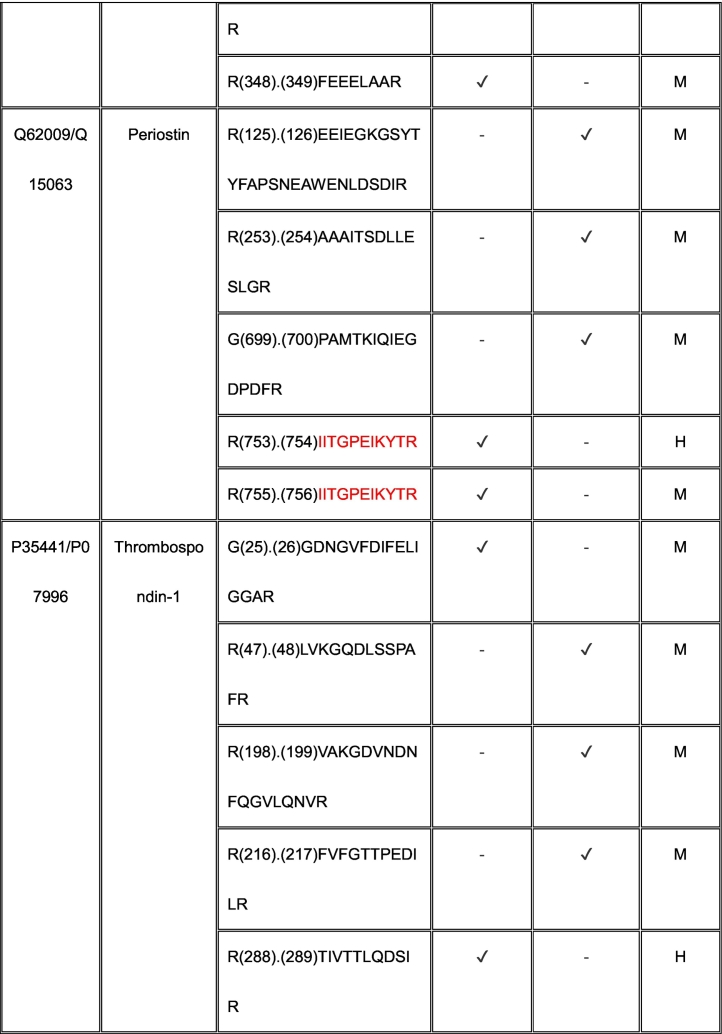
Shared ADAMTS7 and ADAMTS12 substrates identified by TAILS. Cleavage sites shown in column 3 [as amino acid (residue number) cleavage site (.) amino acid (residue number)] were determined from the sequences of N-terminally labeled internal peptides having statistically significant higher abundance in protease-treated medium than the “no-protease” controls. MS data was searched against human (H) and mouse (M) proteins in UniProtKB. Because ADAMTS7 and ADAMTS12 are secreted proteases, only peptides arising from secreted/ECM proteins or the extracellular/lumenal regions of transmembrane proteins were considered. Sequences of peptides in red were identical in human and mouse proteomes, and thus could have arisen from either species. Cleavages and peptide sequences indicating proteolysis at sites with proximate locations in human and mouse sequences are shown in italics; identical cleavage sites of ADAMTS7 and ADAMTS12 in agrin and collagen alpha-2(I) chain are in bold.

The ADAMTS7 TAILS analysis identified nine substrates overlapping with the previous iTRAQ-TAILS analysis by Colige et al., but only one identical cleavage site, also in agrin. However, only 4 overlapping substrates were found between the ADAMTS7 degradome previously identified by Colige et al. [26] and the ADAMTS12 degradome established here.

**Fig. 7:**
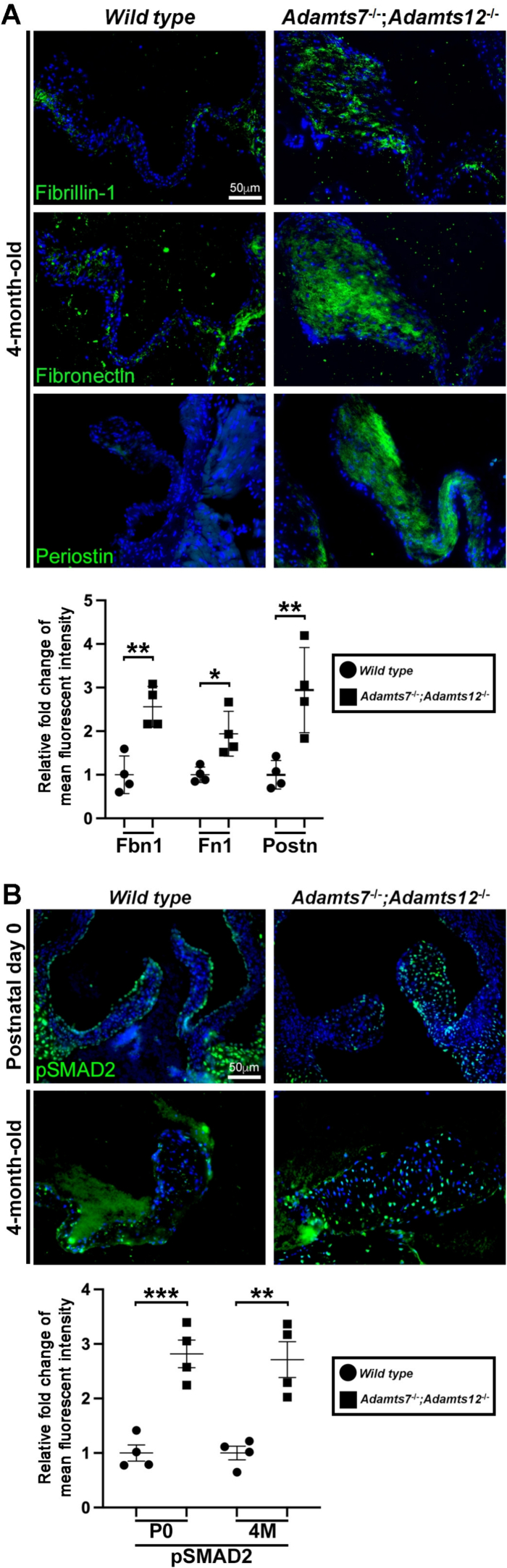
Verified targets of ADAMTS7 and ADAMTS12. **A**. Increased FBN1, FN1, and periostin staining in 4-month-old *Adamts7*^−/−^;*Adamts12*^−/−^ aortic valves. **B.** pSMAD2 staining, indicative of aberrant TGFβ staining, is increased in newborn and adult *Adamts7*^−/−^;*Adamts12*^−/−^ aortic valve leaflets. n=4 mice of each genotype, with two technical replicates for each genotype and time point. Error bars represent SEM. A paired Student t-test was used for statistical analysis. *p ≤ 0.05, **p ≤ 0.01, ***p ≤ 0.001.

We analyzed selected identified ADAMTS7 and ADAMTS12 substrates immunohistochemically in *Adamts7*^−/−^;*Adamts12*^−/−^ and wild type hearts. Consistent with their identification as substrates, *Adamts7*^−/−^;*Adamts12*^−/−^ adult aortic valves had increased staining of fibrillin-1, fibronectin and periostin (Fig. 7A). Because versican binds TGFβ [28], E18.5 valves were stained with an antibody to pSMAD2 as a readout for TGFβ signaling and revealed considerable increase in nuclear pSMAD2 staining of valve interstitial cells throughout the newborn and adult valve leaflet (Fig. 7B).

## 4 Discussion

Congenital heart defects affect approximately 1 % of live births and valve defects, particularly thickened and immature valves, occur in about 30 % of cardiovascular malformations [29]. In adults, myxomatous valve degeneration accounts for significant mortality and morbidity in the aging population. The altered hemodynamics of defective valves initially stimulates adaptive ventricular responses, but once compensatory mechanisms are exhausted, cardiac function declines, with high morbidity and mortality, which frequently necessitate invasive or surgical solutions [30]. Deformed and prosthetic valves are more susceptible to infective endocarditis, an additional clinical challenge in this population. A fundamental understanding of valve components, structure and homeostasis therefore has high significance for human valvular disease.

Aortic valve leaflet extracellular matrix (ECM) has a well-defined architecture reflecting functional adaptation to repeated cycles of compression and tension on the aortic and ventricular aspects of valves, respectively. Elastic fibers are located on the tension (ventricular) side of the aortic valve and abundant collagen and proteoglycan are present on the compression (aortic) side, with an intervening collagen-rich ECM. Aortic and pulmonic valves usually have three semilunar leaflets, which arise from endocardial cushions soon after the heart tube folds. The cushions contain abundant primordial glycosaminoglycan and fibronectin-rich ECM that is remodeled and replaced by the trilaminar adult ECM architecture [31]. Valve anomalies, whether of developmental or adult origin, are usually characterized by altered valve architecture, typically with abnormal ECM and result in aortic stenosis and/or regurgitation [32,33]. Prior work in mice as well as human gene mutations has identified distinct roles for several ADAMTS proteases (e.g., ADAMTS1, ADAMTS5, ADAMTS9, ADAMTS19) in the heart valves [34]. These observations, together with the present study, underscore the pivotal role of proteases in development and maintenance of heart valve ECM.

Here, we describe a mouse model of myxomatous degeneration affecting the aortic, pulmonic, mitral, and tricuspid valves with echocardiographic evidence for aortic regurgitation and stenosis in the absence of left ventricular hypertrophy, but with significantly reduced systolic function and increased chamber dimensions. The mutant valves showed the histologic hallmarks of myxomatous degeneration [35,36] and had evident histologic anomalies at birth, suggesting that myxomatous degeneration may be a continuation of dysregulation initiated during the embryonic period. Whether or not occult developmental defects precede myxomatous change in adult humans is presently unclear.

Consistent with a suspected role for ADAMTS7 and ADAMTS12 in matrix remodeling, we observed changes characteristic of myxomatous degeneration in double knockout mice mutant aortic valve ECM. We sought the mechanistic basis for these changes using degradomics. Prior searches for human ADAMTS7 substrates have used human skin fibroblasts, endothelial cells or aortic smooth muscle cells to provide substrate libraries [7,26], whereas substrates were not previously sought for ADAMTS12. Accordingly, we undertook parallel degradomic analysis of ADAMTS7 and ADAMTS12 using mouse embryonic fibroblasts, which provided a VIC-similar substrate library determined by secretome analysis.

We defined ADAMTS7 activity against a mixed library of human and mouse proteins (seeking substrates of both human and mouse origin), using duplexed dimethyl TAILS. Among ADAMTS7 and ADAMTS12 overlapping substrates (Table 1), we observed abnormal collagen I, fibronectin, periostin and thrombospondin-1 staining in the double mutant valves. However, few cleavage sites were identical, and in addition, distinct substrates of each protease were identified. The lack of valve defects in the single mutant mice suggests that transcriptional adaptation has a significant role in aortic valve ECM maintenance by these proteases. This, together with past work on the ADAMTS7 degradome shows that the substrate repertoire of ADAMTS7 and ADAMTS12 is broad, and several substrates are cleaved at multiple sites, suggesting that the observed myxomatous valve phenotype likely results from impaired turnover of multiple proteins and proteoglycans. ECM has a regulatory impact on cellular signaling by sequestration or mobilization of various growth factors, as well via adhesion receptors. As one example, we observed activation of the canonical TGFβ signaling pathway. Versican, periostin, fibronectin and thrombospondin, substrates whose staining was increased in the mutant valves are all associated with TGFβ-regulation via roles in binding or activation [28,[37], [38], [39]].

Colige et al. identified latent TGFβ-binding proteins as ADAMTS7 substrates [26], which the present study did not, potentially due to the different substrate library used. The elicited degradomes also suggest complex effects on proteolytic enzymes, with proteases BMP1, cathepsin B, cathepsin L1, carboxypeptidase E, insulin-degrading enzyme, MMP2, MMP21, PC5/6, HtrA1 as well as protease inhibitors (TIMP-1, PAI-1, serpin H1, inter-alpha-trypsin inhibitor) or enhancers (PCPE-1) identified as potential ADAMTS7 and ADAMTS12 substrates. This complex substrate profile and secondary impact on proteases is in sharp contrast to ADAMTS13, which appears to have a single substrate, von Willebrand factor [40]. Among the substrates, periostin is a 90 kDa matricellular protein which binds to tenascin-C and fibronectin and organizes collagen 1, each identified as ADAMTS12 substrates. Several collagens were identified as substrates, as well as others that affect collagen biosynthesis (e.g., BMP-1), fibril assembly (e.g., decorin, fibronectin, thrombospondin 1) or proteolysis (e.g., MMP2). Proteoglycans (biglycan, decorin, agrin and perlecan) and basement membrane components (collagen a4(IV), laminin β1 and γ1 subunits) were also identified.

There is a current dearth of mouse models of valvular dysfunction, which are significant health challenges in older individuals and occur in 2–3 % of the population [41]. Among mice with aortic valve myxomatous change and phenotypes relevant to *Adamts7*^−/−^;*Adamts12*^−/−^ adult aortic valves are those with *Col1a2*, *Postn* and *Krox20* mutations [42,43]. *Postn*-Cre mediated *Ctnnb1* deletion led to chondrogenesis in valve leaflets [44], *Eln*^+/−^ mice have cartilage nodules in the valve annulus [45]. Homozygous mouse mutations in *Galnt1* mice [46], reflecting an impact on mucins, have enlarged aortic, pulmonary and AV valves, and are relevant to the present work, since ADAMTS7 and ADAMTS12 have a well conserved mucin module within which the proteoglycan chains are attached, and also show abnormal versican turnover.

In conclusion, the numerous prospective substrates identified by N-terminomics in this and prior studies [7,26] suggest that ADAMTS7 and ADAMTS12 have a broad role in ECM proteostasis and activity against extracellular regions of transmembrane proteins. Their co-expression in valve and transcriptional adaptation suggests that they work cooperatively, together constituting a vital mechanism for maintaining valve ECM proteostasis and protecting valvular function.

There were several limitations of this study. Reliable reagents for ADAMTS7 and ADAMTS12, particularly primary antibodies and purified proteins, are not readily available, so protein localization was not done. Since only the double knockout mouse exhibited a phenotype, it was not possible to determine which of the two genes or which of their substrates was a principal contributor to the phenotype. In addition, TAILS, being a shotgun-proteomics type approach has all the inherent drawbacks of that approach, i.e., peptides that are too small, too large, or carry modifications of unknown mass are not detected. The ECM library provided by MEFs, while overlapping considerable with the valve matrisome, was not identical to it, so one or more valve-relevant substrates could have been missed.

Despite these limitations, the study uncovered several new insights on cardiac development and the potential underlying mechanisms. These include discovery of a novel role for ADAMTS7 and ADAMTS12 in valve matrix proteostasis, resulting from their co-expression during cardiac valve development, identification of novel substrates and comparison of their proteolytic activities. In addition to potential mechanistic insights into myxomatous valve disease in humans, potential translational significance of the work lies in the current consideration of ADAMTS7 as a drug target in atherosclerosis. The current work raises the possibility of disturbance of valve ECM by ADAMTS7 inhibitors if they also block ADAMTS12 activity. The availability of new substrates and cleavages for ADAMTS12 now permits future testing of ADAMTS7 inhibitor specificity.

## CRediT authorship contribution statement

**Timothy J. Mead:** Writing – review & editing, Writing – original draft, Supervision, Methodology, Investigation, Funding acquisition, Formal analysis, Data curation, Conceptualization. **Sumit Bhutada:** Methodology, Formal analysis, Data curation. **Niccolò Peruzzi:** Methodology, Investigation, Formal analysis, Data curation. **Janet Adegboye:** Methodology, Investigation, Formal analysis. **Deborah E. Seifert:** Methodology, Investigation, Data curation. **Elisabeth Cahill:** Methodology, Investigation, Data curation. **Jeanne Drinko:** Methodology, Investigation, Formal analysis, Data curation. **Eoin Donnellan:** Methodology, Investigation. **Anu Guggiliam:** Methodology, Investigation, Data curation. **Zoran Popovic:** Methodology, Formal analysis, Data curation. **Brian Griffin:** Supervision. **Karin Tran-Lundmark:** Supervision, Methodology, Investigation, Formal analysis, Data curation. **Suneel S. Apte:** Supervision, Project administration, Funding acquisition, Formal analysis, Conceptualization, Writing – review & editing.

## Declaration of Generative AI and AI-assisted technologies in the writing process

The authors did not use generative AI or AI-assisted technologies in the development of this manuscript.

## Funding

This work was supported by the Allen Distinguished Investigator Program, through support made by The Paul G. Allen Frontiers Group and the American Heart Association (17DIA33820024 to S.S.A.), NIH NHLBI (HL156987 to T.J.M.) and from the 10.13039/501100003793Swedish Heart-Lung Foundation (20230093 to K.T.L.), the 10.13039/501100004359Swedish Research Council (2022-00683 to K.T.L.), the Skane County Council and the 10.13039/501100004063Knut and Alice Wallenberg Foundation (2022-Projekt0168 to K.T.L). The Fusion Lumos instrument was purchased via an NIH shared instrument grant, 1S10OD023436-0.

## Declaration of competing interest

The authors declare the following financial interests/personal relationships which may be considered as potential competing interests: Timothy Mead reports financial support was provided by 10.13039/100000002NIH. Timothy Mead reports a relationship with 10.13039/100000002National Institutes of Health that includes: funding grants. If there are other authors, they declare that they have no known competing financial interests or personal relationships that could have appeared to influence the work reported in this paper.

## Appendix A Supplementary data

Supplementary material Image 1
