# Supplementary material for: ADAMTS7, a target in atherosclerosis, cooperates with its homolog ADAMTS12 to protect against myxomatous valve degeneration
Source: J Mol Cell Cardiol Plus. 2025 Feb 22;11:100288. doi: 10.1016/j.jmccpl.2025.100288 (PMC11925103; doi:10.1016/j.jmccpl.2025.100288)
Supplement: Supplementary file 1 — Supplementary material [file mmc1.docx]

**MANUSCRIPT SUPPLEMENT**

**ADAMTS7, a target in atherosclerosis, cooperates with its homolog ADAMTS12 to protect against myxomatous valve degeneration**

Timothy J. Mead, Sumit Bhutada, Niccolò Peruzzi, Janet Adegboye, Deborah E. Seifert, Elisabeth Cahill, Jeanne Drinko, Eoin Donnelin, Anu Guggiliam, Zoran Popovic, Brian Griffin, Karin Tran-Lundmark, Suneel S. Apte

This supplement contains:

Figures S1-S9

Table S1-S5

**
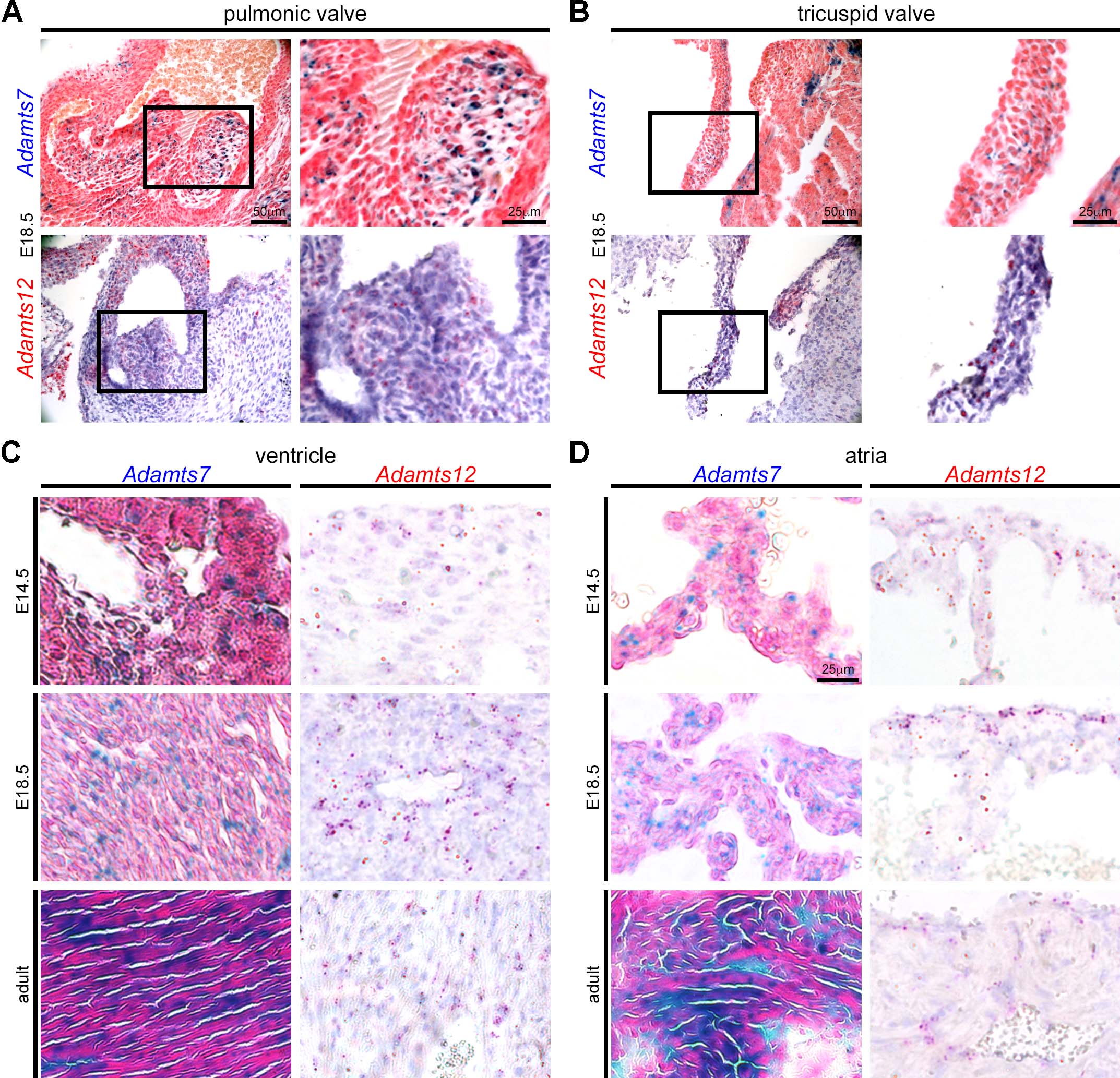
**

**Figure S1. *Adamts7* and *Adamts12* are coordinately expressed in pulmonic valves, tricuspid valves, ventricular myocardium and atrial myocardium during development and adulthood. A-B**. *Adamts7* and *Adamts12* expression is revealed in embryonic and adult mouse hearts by LacZ (blue) and RNA in situ hybridization (red), respectively. Both genes are expressed in pulmonic and tricuspid valves during development and maturation. **C-D**. *Adamts7* and *Adamts12* expression is noted in embryonic and adult ventricular (C) and atrial (D) myocardium. E, embryonic day; PND, postnatal day. n$=$4 biological replicates. Two technical replicates were performed for each time point and stain.

**
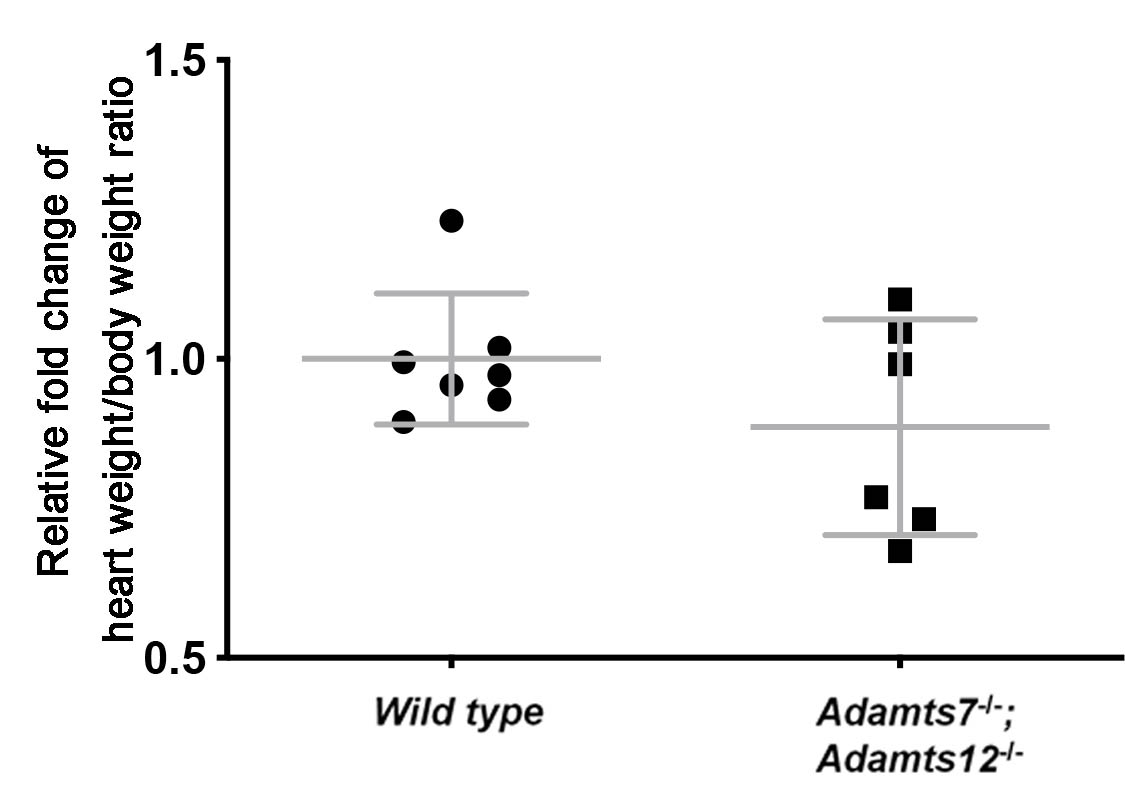
**

**Figure S2. Normal heart weight/body weight ratio of *Adamts7*^-/-^;*Adamts12*^-/-^ mice.** 18 month-old *Adamts7*^-/-^;*Adamts12*^-/-^ mice have no change in heart weight/body weight ratio as compared to age-matched controls. n=7 wild type, 6 *Adamts7*^-/-^;*Adamts12*^-/-^ biological. Error bars represent SEM. Two technical replicates were performed for each time point. Unpaired Student t-test. *p ≤ 0.05.

**
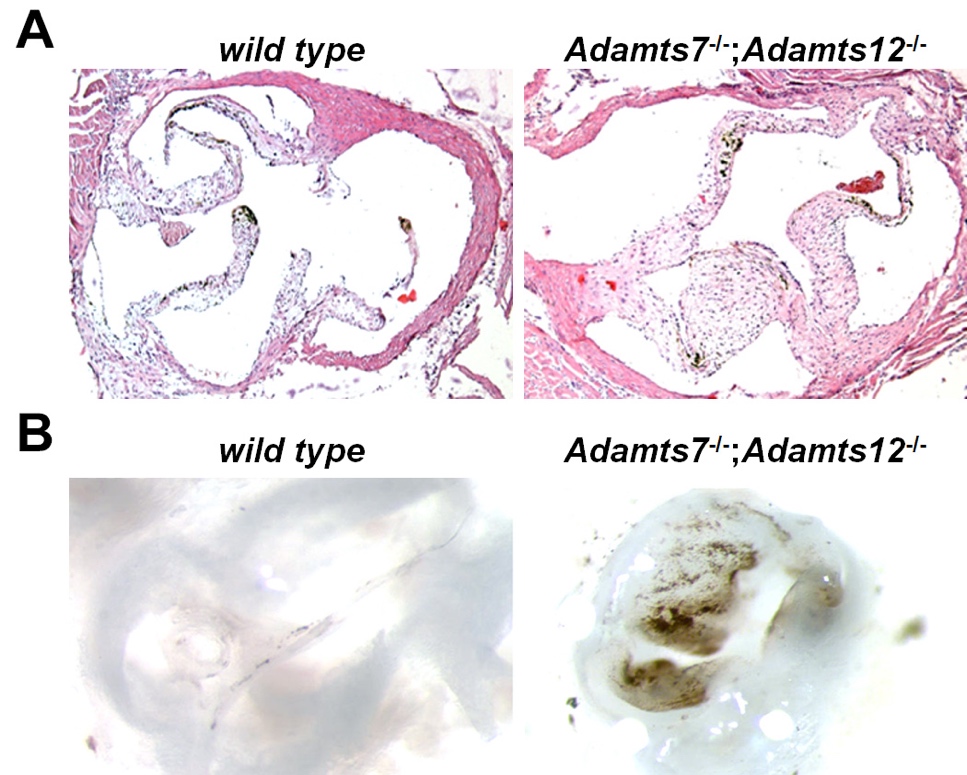
**

**Figure S3. Enhanced pigmentation in *Adamts7*^-/-^;*Adamts12*^-/-^ aortic valves. A.** Hematoxylin and eosin-stained transverse sections of 6-month-old aortic roots showed enlarged valves with prominent melanoctyes (black). **B.** Whole mount photos of freshly-excised aorta and aortic valves showed increased pigmentation. n$=$5 biological replicates and two technical replicates for wholemount and histology of each genotype.


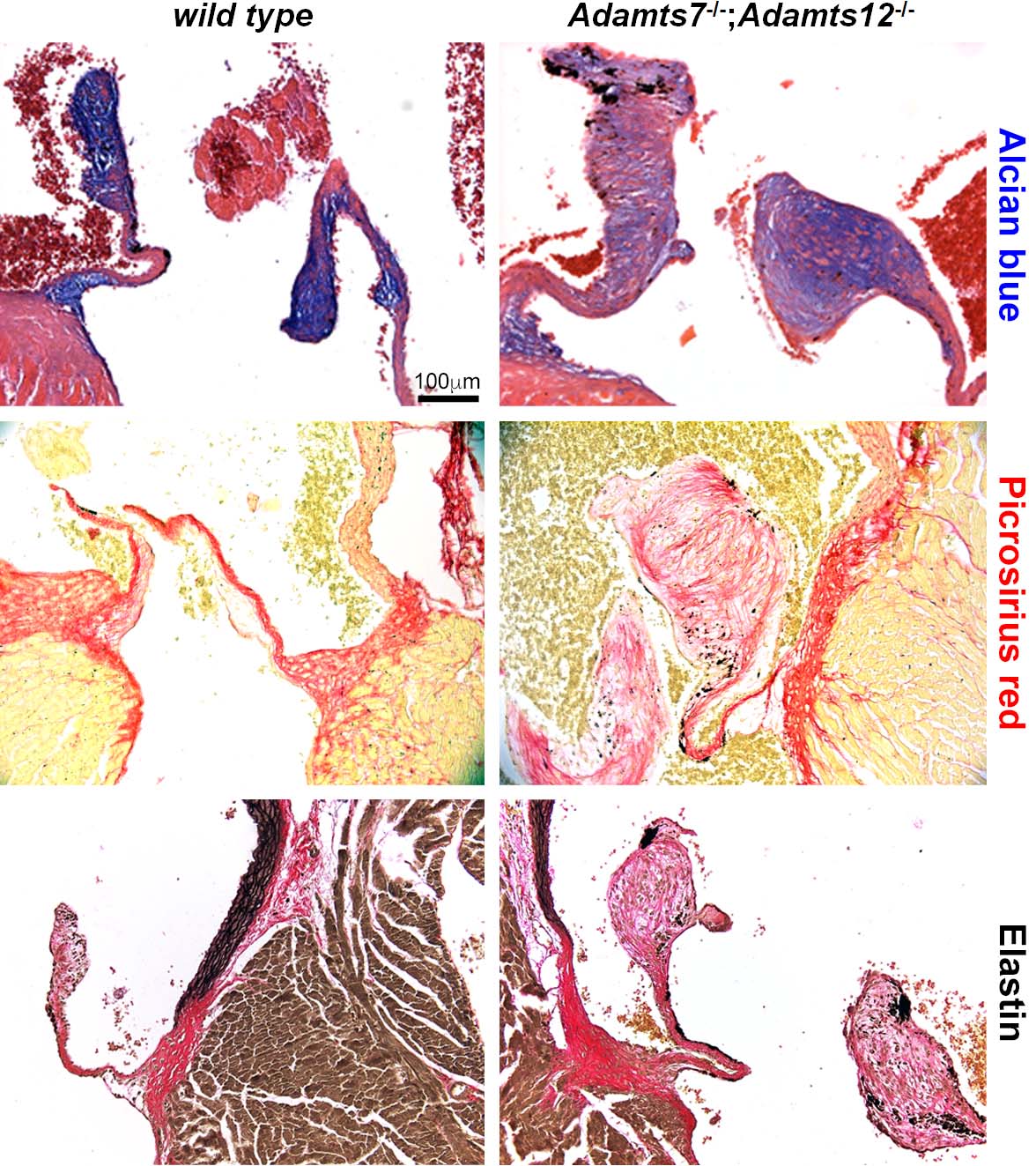


**Figure S4. Disorganized collagen, proteoglycan and elastin in *Adamts7*^-/-^;*Adamts12*^-/-^ aortic valves.** *Adamts7*^-/-^;*Adamts12*^-/-^ aortic valves had expanded proteoglycan Alcian blue staining, and disorganized collagen on picrosirius red staining. Elastin staining was disrupted in the ventricularis layer in *Adamts7*^-/-^;*Adamts12*^-/-^ aortic valves. n$=$4 biological replicates.Two technical replicates were performed for each genotype and stain.

**
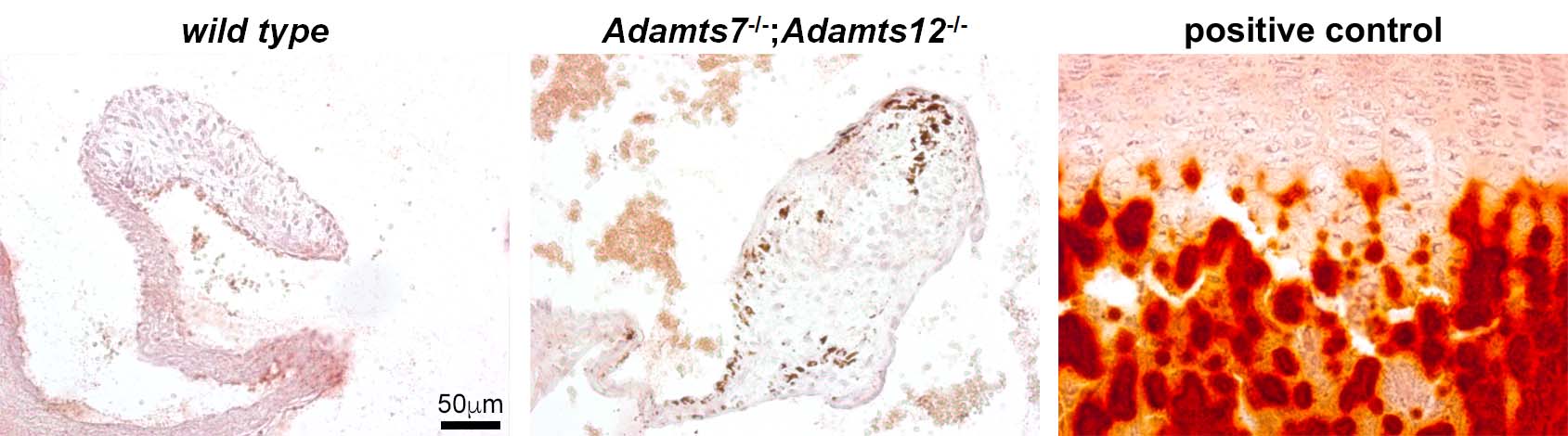
**

**Figure S5. No calcification was evident in *Adamts7*^-/-^;*Adamts12*^-/-^ aortic valves.** Like wild type aortic valves, *Adamts7*^-/-^;*Adamts12*^-/-^ valves showed no calcification as shown by lack of alizarin red S staining. Staining of 18.5 day embryo femur with Alizarin red S is shown as a positive control (red). n$=$4 biological replicates. Two technical replicates were performed for each genotype.

**
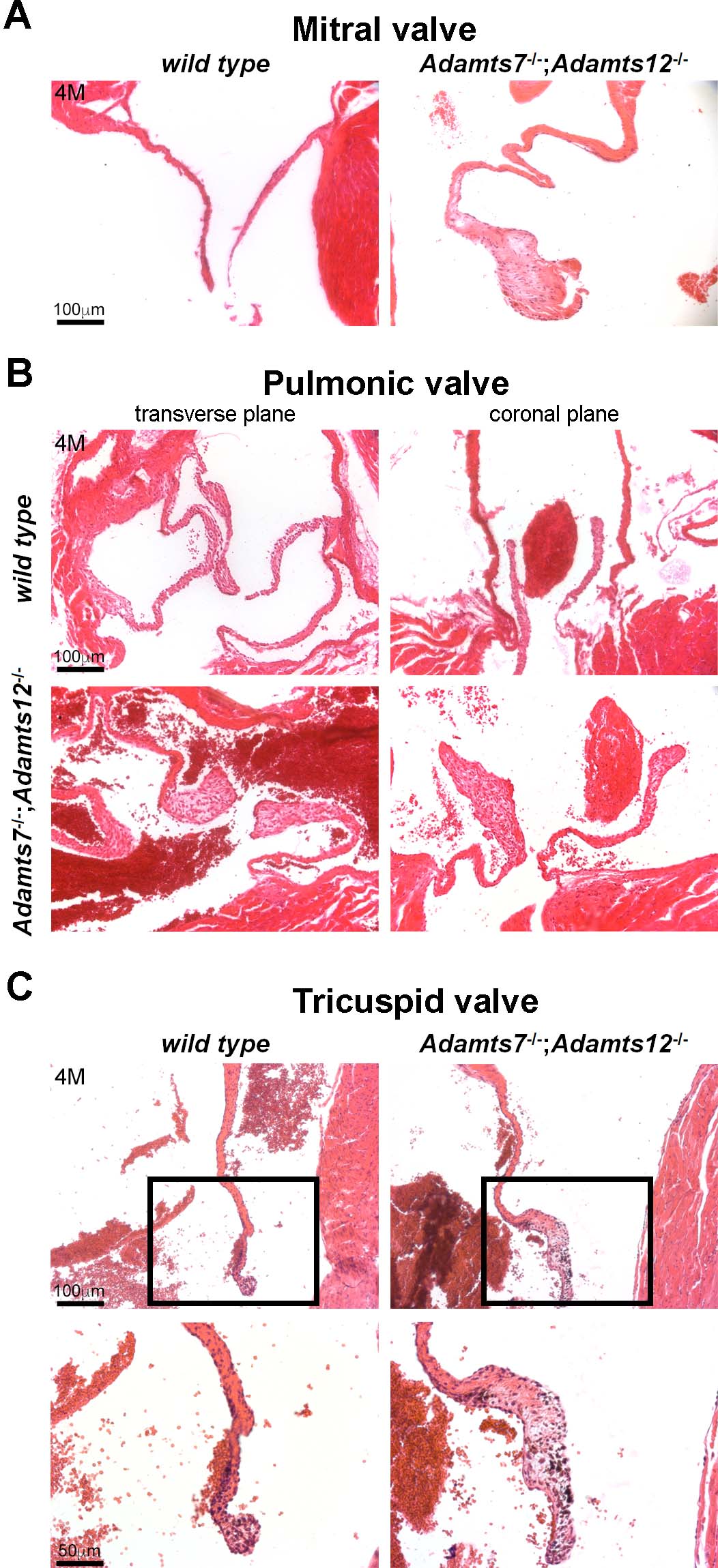
**

**Figure S6. Adult *Adamts7*^-/-^;*Adamts12*^-/-^ heart valves are enlarged.** Hematoxylin and eosin-stained sections of *Adamts7*^-/-^;*Adamts12*^-/-^ mitral, pulmonic and tricuspid heart valves show cusp enlargement relative to wild type. n$=$4 biological replicates. Two technical replicates were performed for each genotype.

**
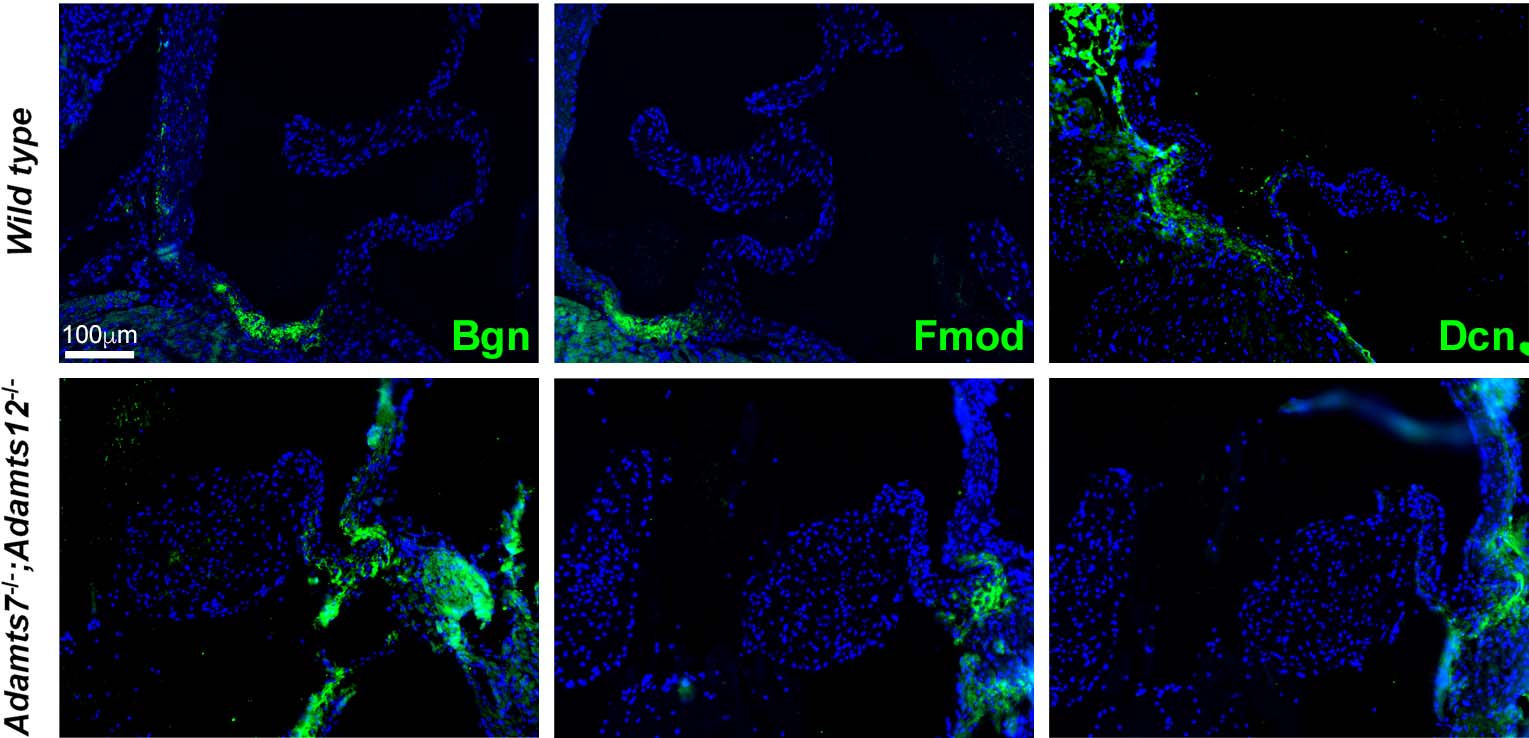
**

**Figure S7. No change in small leucine-rich proteoglycan staining in *Adamts7*^-/-^;*Adamts12*^-/-^ aortic valves.** We observed comparable staining for bigylcan (Bgn), fibromodulin (Fmod) and decorin (Dcn) in aortic valve annulus (green) and no staining in 4-month-old wild type and *Adamts7*^-/-^;*Adamts12*^-/-^ valve leaflets**.** Sections are counterstained with DAPI (blue). n$=$4 biological replicates and two technical replicates of aortic valves for each genotype and stain.

**
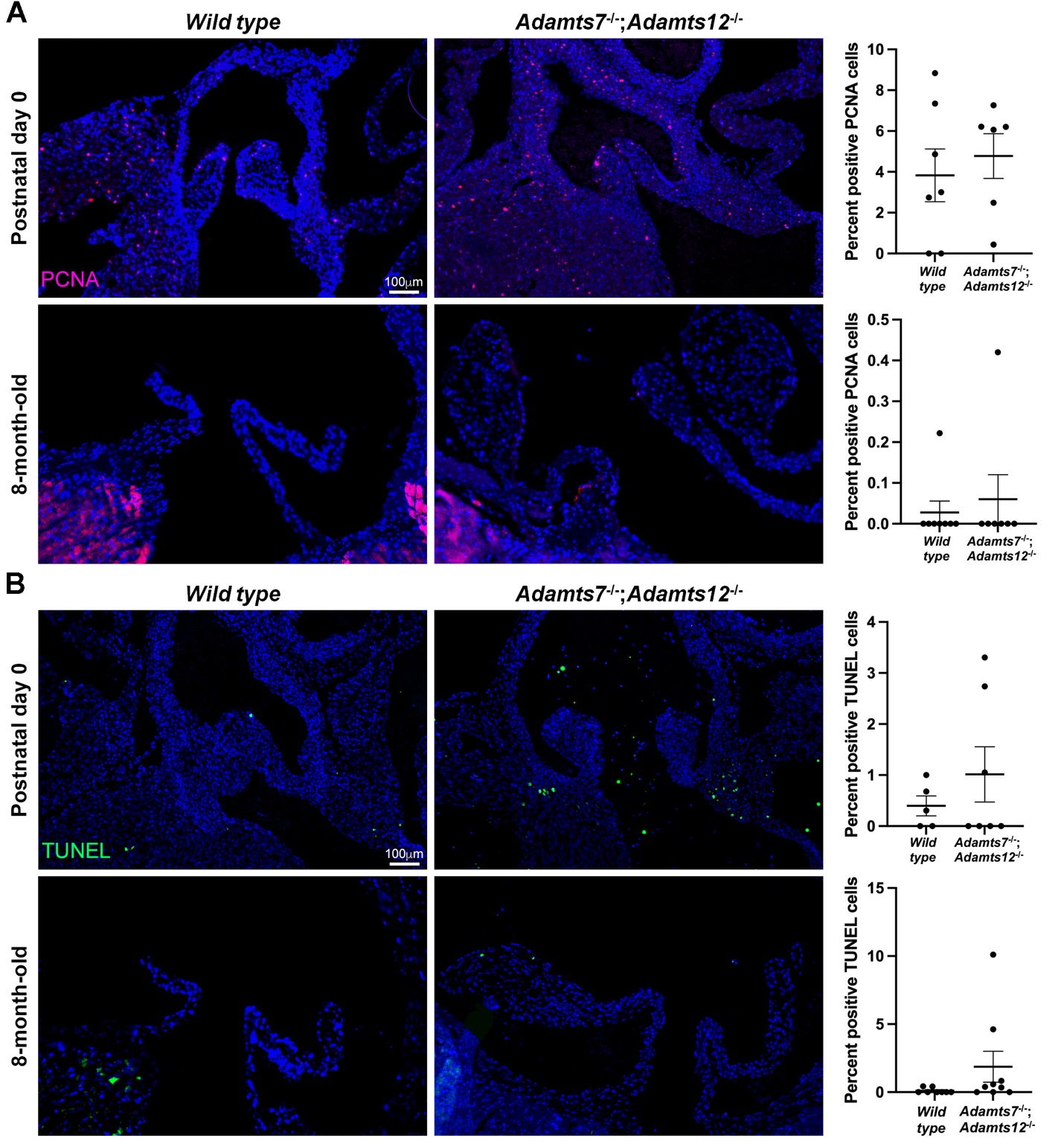
**

**Figure S8. *Adamts7*^-/-^;*Adamts12*^-/-^ aortic valve leaflets have comparable cell proliferation and cell death as wild type. (A)** Cell proliferation was quantified after PCNA immunostaining in aortic valve leaflets. P0: n=7 wild type, 6 *Adamts7*^-/-^;*Adamts12*^-/-^; 8 month: n=8 wild type, 7 *Adamts7*^-/-^;*Adamts12*^-/-^ . Two technical replicates of aortic valves for each genotype. Error bars represent SEM. An unpaired Student t-test was used. **(B)** Cell death was visualized using the TUNEL assay in aortic valve leaflets**.** P0: n=5 wild type, 7 *Adamts7*^-/-^;*Adamts12*^-/-^; 8 month: n=8 wild type, 9 *Adamts7*^-/-^;*Adamts12*^-/-^. Staining was done twice for each genotype. Error bars represent SEM. An unpaired Student t-test was applied.

**
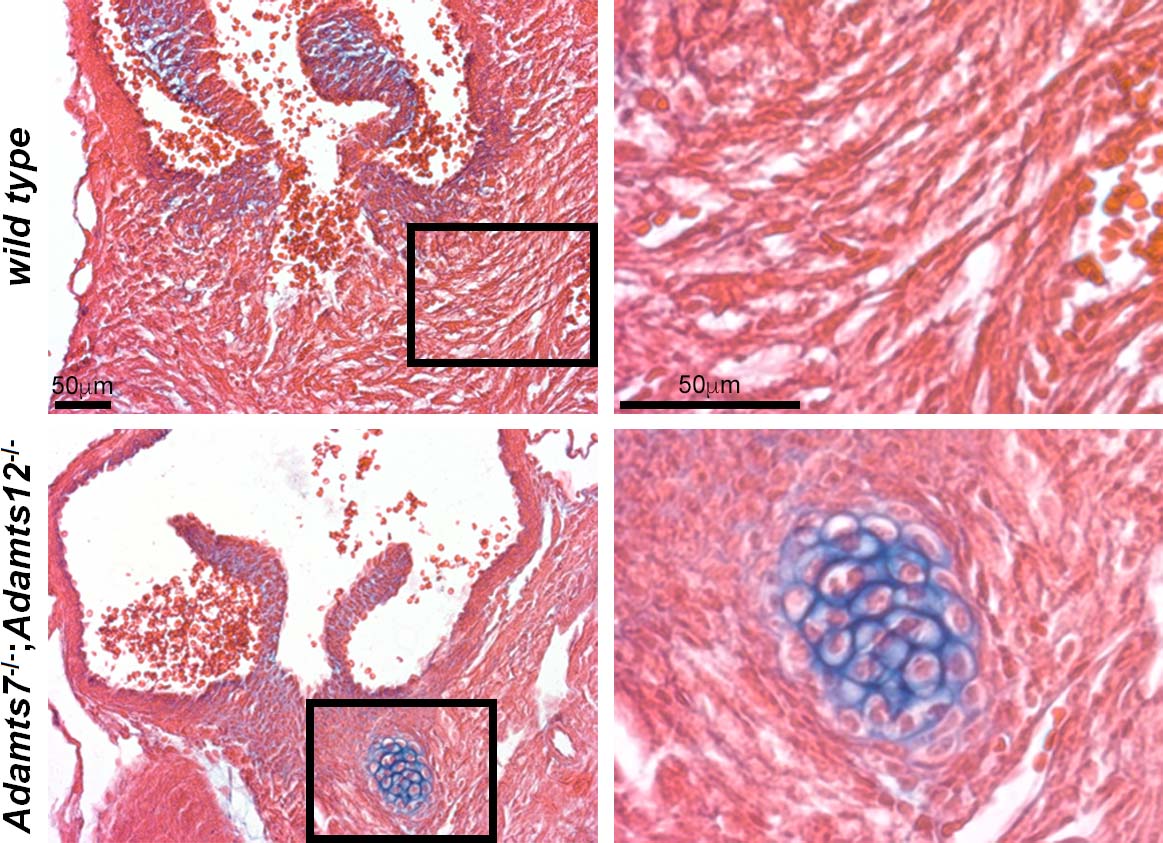
**

**Figure S9. Some *Adamts7*^-/-^;*Adamts12*^-/-^ hearts have ectopic cartilage nodules.** Alcian blue-stained sections showed ectopic cartilage nodules in myocardium in the vicinity of the valvular annulus of 4 of 19 4-month-old *Adamts7*^-/-^;*Adamts12*^-/-^ hearts. n=12 wild type, 19 *Adamts7*^-/-^;*Adamts12*^-/-^ biological replicates and two technical replicates of hearts of each genotype.

**Table S1. Quantitative real-time PCR primers.**

| *Adamts7* | Forward: 5'-GGAGTGAGGACCCAGATAAGTA-3' |
| --- | --- |
|  | Reverse: 5'-CGTGCATAGGTGAAGGTAGTG-3' |
| *Adamts12* | Forward: 5'-CCAAAGGTGCGAGGGATATAAG-3' |
|  | Reverse: 5'-ACCCTCCGTTGAGGTAGTATT-3' |
| *Gapdh* | Forward: 5’-TGGAGAAACCTGCCAAGTATGA-3’ |
|  | Reverse: 5’-CTGTTGAAGTCGCAGGAGACA-3’ |

**Table S2. Antibodies used for immunofluorescence.**

| **Antibody** | **Product #** | **Source** | **Dilution** |
| --- | --- | --- | --- |
| anti-Versican (VC) |  | doi: 10.1074/jbc.M114.573287 | 1:500 |
| anti-DPEAAE | PA1-1748A | Thermo Fisher Scientific | 1:400 |
| Anti-aggrecan | AB1031 | Millipore | 1:400 |
| HABP | #385911 | Millipore | 1:100 |
| Anti-CLP | 9/30/8-A4-c | DSHB | 1:100 |
| Anti-COMP | PC-140 | Kamiya | 1:100 |
| Anti-Fibrillin-1 |  | From Dr. Dieter Reinhardt | 1:1000 |
| Anti-Fibronectin | AB2033 | Abcam | 1:400 |
| Anti-Periostin |  | PMID: 10404027 | 1:500 |
| anti-pSMAD2 | #3101 | Cell Signaling | 1:200 |
| anti-ADAMTS7 | AB45044 | Abcam | 1:100 |
| anti-ADAMTS12 | 24934-1-AP | Proteintech | 1:50 |
| anti-biglycan | LF-159 | From Dr. Larry Fisher (NIH) | 1:200 |
| anti-fibromodulin | LF-150 | From Dr. Larry Fisher (NIH) | 1:200 |
| anti-decorin | LF-114 | From Dr. Larry Fisher (NIH) | 1:200 |
| Anti-PCNA | 2586 | Cell Signaling | 1:200 |

**Table S3. Observed incidence of aortic valve regurgitation over time.**

|  | **4-month-old** | **6-month-old** | **12-month-old** |
| --- | --- | --- | --- |
| ***Wild type*** | 0/10 (0%) | 0/10 (0%) | 0/10 (0%) |
| ***Adamts7*^-/-^;*Adamts12*^-/-^** | 3/10 (30%) | 3/13 (23%) | 9/15 (60%) |

**Table S4. Putative ADAMTS7 substrates identified by TAILS**. Cleavage sites shown in column 3 [as amino acid (residue number).(cleavage site) (residue number) amino acid] were inferred from the sequences of N-terminally labeled internal peptides (TAILS peptides, sequence following .) having statistically significant higher abundance in protease-treated medium than the controls (no protease). Peptides were identified using duplex dimethyl TAILS (n=3 ADAMTS7, n=3 control). MS data was searched against human (H) and mouse (M) proteins in UniProtKB. Because ADAMTS7 is a secreted protease, only peptides arising from secreted/ECM proteins or the extracellular/lumenal regions of transmembrane proteins were considered. Sequences of peptides in red were identical in human and mouse proteomes, hence could have arisen from either species.

| Uniprot Accession | Protein name | TAILS peptide | (log2): ADAMTS7 / Control | Adjusted P-Value: ADAMTS7 / Control | Species |
| --- | --- | --- | --- | --- | --- |
| A2ASQ1 | Agrin | K(1745).(1746)SVGDLETLAFDGR | 1.02 | 0.01935 | M |
| P02771 | Alpha-fetoprotein | L(365).(366)AVSVILR | 4.35 | 0.00781 | H |
| P61769 | Beta-2-microglobulin | C(45).(46)YVSGFHPSDIEVDLLKNGER | 3.94 | 0.00564 | H |
| P21810 | Biglycan | R(172).(173)KVPKGVFSGLR | 3.26 | 0.00125 | H |
| P16870 | Carboxypeptidase E | R(41).(42)RLQQEDGISFEYHR | 1.46 | 0.03265 | H |
| P10605 | Cathepsin B | C(59).(60)GTVLGGPKLPGR | 1.41 | 0.03356 | M |
|  |  | N(298).(299)SWNLDWGDNGFFKILR | 1.50 | 0.02532 | M |
|  |  | C(319).(320)GIESEIVAGIPR | 1.58 | 0.01391 | M |
| P06797 | Cathepsin L1 | G(194).(195)GLDSEESYPYEAKDGSCKYR | 3.75 | 0.00605 | M |
| P11087/  P02452 | Collagen alpha-1(I) chain | R(674).(675)GVQGPPGPAGPR | 3.52 | 0.00490 | M |
|  |  | R(685).(686)GVQGPPGPAGPR | 3.61 | 0.00521 | H |
|  |  | R(854).(855)GAAGPPGATGFPGAAGR | 4.01 | 0.0078 | M |
|  |  | E(1057).(1058)TGPAGPAGPIGPAGAR | 3.87 | 0.00658 | M |
|  |  | L(1377).(1378)LLQGSNEIELR | 4.52 | 0.00813 | M |
|  |  | L(1388).(1389)LLQGSNEIEIR | 4.55 | 0.00785 | H |
| P02458 | Collagen alpha-1(II) chain | G(888).(889)AQGPPGATGFPGAAGR | 3.97 | 0.00686 | H |
| P08121/  P02461 | Collagen alpha-1(III) chain | R(955).(956)GLAGPPGMPGPR | 4.26 | 0.00697 | M |
|  |  | R(956).(957)GLAGPPGMPGPR | 4.35 | 0.00753 | H |
| Q99715/  Q60847 | Collagen alpha-1(XII) chain | N(1275).(1276)TLTGMALNFIR | 4.15 | 0.00894 | H |
|  |  | N(1275).(1276)TLTGMALNFIR | 4.12 | 0.00871 | M |
| Q01149 | Collagen alpha-2(I) chain | A(867).(868)GPQGLLGAPGILGLPGSR | 2.77 | 0.00934 | M |
|  |  | R(984).(985)GEPGPAGSVGPVGAVGPR | 2.79 | 0.00935 | M |
|  |  | R(1073).(1074)SGQPGPVGPAGVR | 3.71 | 0.00717 | M |
|  |  | G(1111).(1112)GGYDFGFEGDFYR | 1.06 | 0.0263 | M |
|  |  | G(1112).(1113)GYDFGFEGDFYR | 1.93 | 0.02129 | M |
| Q9QZR9 | Collagen alpha-4(IV) chain | T(104).(105)GVPGFPGVDGVPGHPGPPGPR | 3.61 | 0.00649 | M |
| P01027 | Complement C3 | R(1582).(1583)KFISHIKCR | 3.72 | 0.00607 | M |
| P10889 | C-X-C motif chemokine 2 | G(27).(28)AVVASELR | 3.80 | 0.0064 | M |
| P11276 | Fibronectin | C(401).(402)TDHAVLVQTR | 3.71 | 0.00617 | M |
|  |  | A(771).(772)TSVNIPDLLPGR | 2.82 | 0.00938 | M |
|  |  | T(772).(773)SVNIPDLLPGR | 1.59 | 0.02223 | M |
|  |  | R(1374).(1375)VTWAPPPSIELTNLLVR | 4.12 | 0.00522 | M |
|  |  | R(1472).(1473)APITGYIIR | 4.56 | 0.00847 | M |
|  |  | N(2169).(2170)VDEEVQIGHVPR | 4.16 | 0.00657 | M |
|  |  | N(2344).(2345)GVNYKIGEKWDR | 4.32 | 0.00843 | M |
| P37889 | Fibulin-2 | E(544).(545)GEEPLIVPEVR | 4.25 | 0.00747 | M |
| Q07797 | Galectin-3-binding protein | R(104).(105)SLGWMVSR | 2.70 | 0.00935 | M |
| P13020 | Gelsolin | T(49).(50)MVVEHPEFLKAGKEPGLQIWR | 2.66 | 0.01018 | M |
| P11142 | Heat shock cognate 71 kDa protein | N(35).(36)RTTPSYVAFTDTER | 4.04 | 0.001118 | H |
|  |  | R(155).(156)QATKDAGTIAGLNVLR | 4.46 | 0.00768 | H |
|  |  | R(301).(302)FEELNADLFR | 4.23 | 0.00891 | H |
| Q06033 | Inter-alpha-trypsin inhibitor heavy chain H3 | R(546).(547)DYIFGNYIER | 4.20 | 0.00887 | H |
| P01042 | Kininogen-1 | L(378).(379)MKRPPGFSPFR | 2.73 | 0.00289 | H |
| P02788 | Lactotransferrin | N(539).(540)SNERYYGYTGAFR | 4.39 | 0.00984 | H |
| P02468/  P11047 | Laminin subunit gamma-1 | N(234).(235)SPVLQEWVTATDIR | 4.22 | 0.00921 | M |
|  |  | N(236).(237)SPVLQEWVTATDIR | 2.28 | 0.00922 | H |
| Q8K3F2 | Matrix metalloproteinase-21 | K(59).(60)YGWSEIPSPKESAGVPVGFTLAQA | 4.11 | 0.00841 | M |
| P14543/  P10493 | Nidogen-1 | R(1017).(1018)QDLGSPEGIAVDHLGR | 2.77 | 0.00935 | H |
|  |  | R(1073).(1074)GNLYWTDWNR | 4.04 | 0.00737 | M |
|  |  | R(1075).(1076)GNLYWTDWNR | 4.07 | 0.00751 | H |
| Q02819 | Nucleobindin-1 | R(348).(349)FEEELAAR | 3.98 | 0.00657 | M |
| Q62009/  Q15063 | Periostin | R(753).(754)IITGPEIKYTR | 4.49 | 0.0081 | H |
|  |  | R(755).(756)IITGPEIKYTR | 4.46 | 0.00776 | M |
|  |  | R(369).(370)MAPTEMVIDR | 2.87 | 0.00935 | M |
| P22777 | Plasminogen activator inhibitor 1 |  |  |  |  |

**Table S5. Putative ADAMTS12 substrates identified by TAILS**. Cleavage sites shown in column 3 [as amino acid (residue number).(cleavage site) (residue number) amino acid] were determined from the sequences of N-terminally labeled internal peptides (TAILS peptides, sequence following the cleavage sites) having statistically significant higher abundance in protease-treated medium than the “no-protease” controls. MS data from dimethyl (n=3 ADAMTS12, n=3 control) and 8-plex iTRAQ-TAILS (n=4 ADAMTS12, n=4 control) was searched against human (H) and mouse (M) proteins in UniProtKB. To determine statistical significance, the Student t-test was applied for dimethyl-TAILS and ANOVA was applied for iTRAQ-TAILS. Because ADAMTS12 is a secreted protease, only peptides arising from secreted/ECM proteins or the extracellular/lumenal regions of transmembrane proteins were considered. Sequences of peptides in red were identical in human and mouse proteomes, hence could have arisen from either species.

| Uniprot Accession | Protein name | TAILS peptide | Label | (log2): ADAMTS12 / Control | Adjusted P-Value: ADAMTS12 / Control | Species |
| --- | --- | --- | --- | --- | --- | --- |
| P08253 | 72 kDa type IV collagenase (MMP2) | R(115).(116)KPKWDKNQITYR | Dimethyl | 4.40 | 0.00931 | H |
| P58397 | A disintegrin and metalloproteinase with thrombospondin motifs 12 | R(1336).(1337)RVECSTQMDSDCAAIQRPDPAKR | Dimethyl | 4.32 | 0.00904 | H |
|  |  | R(1337).(1338)VECSTQMDSDCAAIQRPDPAKR | Dimethyl | 4.08 | 0.00681 | H |
|  |  | R(1387).(1388)EIQCVDSR | Dimethyl | 4.13 | 0.00985 | H |
|  |  | R(82).(83)DLDGSEDWVYYR | iTRAQ | 1.37 | 0.048884 | H |
| O00468 | Agrin | K(1863).(1864)SAGDVDTLAFDGR | iTRAQ | 1.82 | 0.048884 | H |
| Q61247 | Alpha-2-antiplasmin | F(418).(419)IMEDTIGVPLFVGSVR | Dimethyl | 4.53 | 0.00758 | M |
| P02765 | Alpha-2-HS-glycoprotein | C(132).(133)DSSPDSAEDVR | Dimethyl/  iTRAQ | 4.04 | 0.00718 | H |
| Q05793 | Basement membrane-specific heparan sulfate proteoglycan core protein (perlecan) | R(624).(625)GMLEPVQKPDVILVGAGYR | iTRAQ | 1.42 | 0.008307 | M |
|  |  | R(998).(999)GDKVTSYGGELR | iTRAQ | 1.97 | 0.009808 | M |
| P15535 | Beta-1,4-galactosyltransferase 1 | H(177).(178)KVAIIIPFR | iTRAQ | 1.72 | 0.009997 | M |
| P01887  P61769 | Beta-2-microglobulin | Y(86).(87)YTEFTPTEKDEYACR | Dimethyl | 4.18 | 0.00105 | H |
|  |  | R(101).(102)VKHASMAEPKTVYWDR | Dimethyl | 4.67 | 0.00630 | M |
| P98063 | Bone morphogenetic protein 1 | R(309).(310)LSKGDIAQAR | iTRAQ | 1.66 | 0.009808 | M |
| P02452/  P11087 | Collagen alpha-1(I) chain | L(1238).(1239)SQQIENIR | Dimethyl | 4.87 | 0.00796 | H |
|  |  | R(482).(483)GFPGADGVAGPKGPSGER | iTRAQ | 1.9 | 0.009808 | M |
|  |  | R(1216).(1217)DLEVDTTLKSLSQQIENIR | iTRAQ | 1.31 | 0.007252 | M |
| Q04857 | Collagen alpha-1(VI) chain | R(935).(936)VLLFSDGNSQGATAEAIEKAVQEAQR | iTRAQ | 1.93 | 0.009808 | M |
|  |  | R(981).(982)VLVTGKTAEYDVAFGER | iTRAQ | 1.33 | 0.007135 | M |
| Q60847 | Collagen alpha-1(XII) chain | R(499).(500)VEDIIKAINTFPYR | iTRAQ | 2.32 | 0.008098 | M |
|  |  | R(756).(757)YRPVSGGESKEVSTPANQR | iTRAQ | 2.05 | 0.009808 | M |
|  |  | R(2236).(2237)LKLSPADGTR | iTRAQ | 2.04 | 0.009808 | M |
| P08123/  Q01149 | Collagen alpha-2(I) chain | R(777).(778)GDGGPPGMTGFPGAAGR | Dimethyl | 4.19 | 0.0078 | H |
|  |  | R(783).(784)GDGGPPGMTGFPGAAGR | Dimethyl | 4.19 | 0.0078 | M |
|  |  | R(984).(985)GEPGPAGSVGPVGAVGPR | Dimethyl | 4.37 | 0.00824 | M |
|  |  | R(1073).(1074)SGQPGPVGPAGVR | Dimethyl | 4.25 | 0.00496 | M |
|  |  | R(456).(457)GLPGSPGNVGPSGKEGPVGLPGIDGR | iTRAQ | 1.05 | 0.006526 | M |
| Q02788 | Collagen alpha-2(VI) chain | R(489).(490)GPQGALGEPGKQGSR | iTRAQ | 2.0 | 0.009808 | M |
|  |  | R(1013).(1014)EKDFDSLAQPSFFDR | iTRAQ | 2.23 | 0.008547 | M |
| P14106 | Complement C1q subcomponent subunit B | R(139).(140)FEKVITNANENYEPR | iTRAQ | 1.93 | 0.009808 | M |
| P01024 | Complement C3 | Q(983).(984)GTPVAQMTEDAVDAER | Dimethyl | 3.10 | 0.00995 | H |
| P28654 | Decorin | R(315).(316)KASYSAVSLYGNPVR | Dimethyl | 2.60 | 0.00181 | M |
|  |  | R(315).(316)KASYSAVSLYGNPVR | iTRAQ | 1.97 | 0.009808 | M |
| Q14126 | Desmoglein-2 | R(242).(243)DGNGEVTDKPVKQAQVQIR | iTRAQ | 1.12 | 0.041046 | H |
| Q62165 | Dystroglycan (alpha) | Q(213).(214)SFSEVELHNMKLVPVVNNR | iTRAQ | 2.0 | 0.009808 | M |
| P11276/  P02751 | Fibronectin | A(279).(280)SAGSGSFTDVR | Dimethyl | 5.10 | 0.00882 | M |
|  |  | R(369).(370)TFYSCTTEGR | Dimethyl | 4.19 | 0.00472 | H |
|  |  | R(1751).(1752)YRVTYSSPEDGIR | Dimethyl | 4.37 | 0.00953 | M |
|  |  | R(479).(480)IGDQWDKQHDLGH | iTRAQ | 1.85 | 0.009956 | M |
|  |  | R(567).(568)TFYQIGDSWEKFVHGVR | iTRAQ | 1.98 | 0.009808 | M |
|  |  | R(1111).(1112)IGFKLGVRPSQGGEAPR | iTRAQ | 1.76 | 0.009808 | M |
|  |  | F(1114).(1115)KLGVRPSQGGEAPR | iTRAQ | 2.01 | 0.009808 | M |
|  |  | N(1185).(1186)PDTGVLTVSWER | iTRAQ | 1.08 | 0.048884 | H |
|  |  | Y(1883).(1884)ALKDTLTSRPAQGVITTLENVSPPR | iTRAQ | 1.2 | 0.007192 | M |
|  |  | R(1929).(1930)ITGYIIKYEKPGSPPR | iTRAQ | 1.01 | 0.041046 | H |
|  |  | Y(1933).(1934)IIKYEKPGSPPR | iTRAQ | 1.18 | 0.04062 | H |
|  |  | R(1956).(1957)SYTITGLQPGTDYKIHLYTLNDNAR | iTRAQ | 1.6 | 0.009808 | M |
| Q62356/  Q12841 | Follistatin-related protein 1 | R(23).(24)SKSKICANVFCGAGR | Dimethyl | 4.66 | 0.00765 | M |
|  |  | R(25).(26)SKSKICANVFCGAGR | Dimethyl | 4.66 | 0.00775 | H |
| Q9Z0L8 | Gamma-glutamyl hydrolase | N(52).(53)YYIAASYVKYIESAGAR | iTRAQ | 1.39 | 0.007933 | M |
| P06396/  P13020 | Gelsolin | M(52).(53)VVEHPEFLKAGKEPGLQIWR | Dimethyl/  iTRAQ | 4.86 | 0.00866 | H |
|  |  | F(174).(175)KHVVPNEVVVQR | iTRAQ | 2.14 | 0.008892 | M |
|  |  | P(403).(404)GLGYLSSHIANVER | iTRAQ | 1.36 | 0.0075 | M |
| P06745 | Glucose-6-phosphate isomerase | R(6).(7)NPQFQKLLEWHR | iTRAQ | 2.04 | 0.009808 | M |
| Q9CZD3 | Glycine--tRNA ligase | F(620).(621)VKELSEALTR | iTRAQ | 2.54 | 0.005264 | M |
| P07901 | Heat shock protein HSP 90-alpha | N(30).(31)TFYSNKEIFLR | iTRAQ | 1.35 | 0.007362 | M |
|  |  | H(184).(185)LKEDQTEYLEER | iTRAQ | 2.25 | 0.008512 | M |
| P08238 | Heat shock protein HSP 90-beta | G(667).(668)FSLEDPQTHSNR | Dimethyl | 4.12 | 0.00958 | H |
| Q61191 | Host cell factor 1 | R(1996).(1997)NEKGYGPATQVR | iTRAQ | 1.97 | 0.009808 | M |
| Q9JHR7 | Insulin-degrading enzyme | R(711).(712)LKAFIPQLLSR | iTRAQ | 1.99 | 0.009808 | M |
|  |  | R(824).(825)TKEQLGYIVFSGPR | iTRAQ | 1.32 | 0.008884 | M |
|  |  | R(847).(848)FIIQSEKPPHYLESR | iTRAQ | 1.89 | 0.009933 | M |
| P47877 | Insulin-like growth factor-binding protein 2 | L(37).(38)FRCPPCTPER | Dimethyl | 4.04 | 0.0045 | M |
| Q61702 | Inter-alpha-trypsin inhibitor heavy chain H1 | L(126).(127)GESAGLVR | iTRAQ | 3.06 | 0.000645 | M |
| P02469 | Laminin subunit beta-1 | R(208).(209)ALDPAFKIEDPYSPR | iTRAQ | 1.73 | 0.009808 | M |
| Q99MN1 | Lysine--tRNA ligase | R(134).(135)ASGGKLIFYDLR | iTRAQ | 1.78 | 0.009808 | M |
|  |  | R(295).(296)IAPELYHKMLVVGGIDR | iTRAQ | 1.95 | 0.009808 | M |
|  |  | H(302).(303)KMLVVGGIDR | iTRAQ | 1.49 | 0.008613 | M |
| P16675 | Lysosomal protective protein | R(341).(342)KALHIPESLPR | iTRAQ | 2.3 | 0.008307 | M |
| P34884 | Macrophage migration inhibitory factor | H(63).(64)SIGKIGGAQNR | iTRAQ | 2.54 | 0.004875 | M |
| P01033 | Metalloproteinase inhibitor 1 (TIMP1) | R(185).(186)HLACLPR | Dimethyl | 4.31 | 0.00516 | H |
| P12025 | Midkine | R(108).(109)YNAQCQETIR | Dimethyl | 4.16 | 0.00427 | M |
| Q9R0E1 | Multifunctional procollagen lysine hydroxylase and glycosyltransferase LH3 | H(616).(617)MKQVGYEDQWLQLLR | iTRAQ | 1.76 | 0.009994 | M |
| Q02818 | Nucleobindin-1 | R(87).(88)ELDFVSHHVR | Dimethyl | 4.0 | 0.00708 | H |
|  |  | R(188).(189)YLESLGEEQR | Dimethyl | 4.36 | 0.006311 | M |
| P62937/  P17742 | Peptidyl-prolyl cis-trans isomerase A | R(19).(20)VSFELFADKVPKTAENFR | iTRAQ | 1.42 | 0.041046 | H |
|  |  | K(131).(132)VKEGMNIVEAMER | iTRAQ | 1.27 | 0.006323 | M |
| Q62009 | Periostin | R(125).(126)EEIEGKGSYTYFAPSNEAWENLDSDIR | iTRAQ | 1.99 | 0.009808 | M |
|  |  | R(253).(254)AAAITSDLLESLGR | Dimethyl | 4.74 | 0.008057 | M |
|  |  | G(699).(700)PAMTKIQIEGDPDFR | iTRAQ | 1.55 | 0.009279 | M |
| P97298 | Pigment epithelium-derived factor | R(223).(224)KTTLQDFHLDEDR | iTRAQ | 2.47 | 0.006228 | M |
|  |  | K(348).(349)PVKLTQVEHR | iTRAQ | 1.25 | 0.006228 | M |
| O75051 | Plexin-A2 | R(308).(309)LLQAAYLAKPGDSLAQAF | iTRAQ | 1.13 | 0.04062 | H |
| Q61398 | Procollagen C-endopeptidase enhancer 1 | R(274).(275)DAVEKESALSPGEDVQR | iTRAQ | 2.43 | 0.006534 | M |
| Q9R0B9 | Procollagen-lysine,2-oxoglutarate 5-dioxygenase 2 | R(411).(412)KIIAPLVTR | iTRAQ | 1.05 | 0.003632 | M |
| Q04592 | Proprotein convertase subtilisin/kexin type 5 (PC5/6) | R(160).(161)GYTGKNIVVTILDDGIER | iTRAQ | 2.25 | 0.008315 | M |
| Q9R118 | Serine protease HTRA1 | R(302).(303)GGKELGLR | iTRAQ | 1.71 | 0.009808 | M |
| P19324 | Serpin H1 | H(261).(262)KLSSLIILMPHHVEPLER | iTRAQ | 1.95 | 0.009808 | M |
| Q9H4F8 | SPARC-related modular calcium-binding protein 1 | F(34).(35)LISDRDPQCNLHCSR | Dimethyl | 4.29 | 0.007139 | H |
| Q80YX1 | Tenascin | R(914).(915)NVKADIDSYR | iTRAQ | 2.6 | 0.004459 | M |
|  |  | R(1674).(1675)LVKLTPGVEYR | iTRAQ | 1.81 | 0.0099 | M |
| P35441 | Thrombospondin-1 | R(216).(217)FVFGTTPEDILR | Dimethyl | 3.12 | 0.00935 | M |
|  |  | R(47).(48)LVKGQDLSSPAFR | iTRAQ | 1.76 | 0.009808 | M |
|  |  | R(198).(199)VAKGDVNDNFQGVLQNVR | iTRAQ | 1.99 | 0.009808 | M |
| P02786 | Transferrin receptor protein 1 | R(109).(110)EEPGEDFPAAR | iTRAQ | 1.21 | 0.048884 | H |
|  |  | R(121).(122)LYWDDLKR | iTRAQ | 1.5 | 0.054481 | H |
|  |  | R(184).(185)LREKLQEEMLQR | Dimethyl | 4.37 | 0.00752 | H |
|  |  | R(570).(571)ALASQLQDSLKDLKAR | iTRAQ | 1.33 | 0.041046 | H |
